# Substituent Effects on NMR Spectroscopy of 2,2-Dimethylchroman-4-one Derivatives: Experimental and Theoretical Studies

**DOI:** 10.3390/molecules25092061

**Published:** 2020-04-28

**Authors:** Daniela Iguchi, Davide Ravelli, Rosa Erra-Balsells, Sergio M. Bonesi

**Affiliations:** 1 CIHIDECAR-CONICET–Departamento de Química Orgánica, Facultad de Ciencias Exactas y Naturales, 3er Piso, Pabellón 2, Ciudad Universitaria, University of Buenos Aires, CP 1428 Buenos Aires, Argentina; daniela.iguchi@fadu.uba.ar (D.I.); erra@qo.fcen.uba.ar (R.E.-B.); 2 Design and Chemistry of Macromolecules Group, Institute of Technology in Polymers and Nanotechnology (ITPN), UBA-CONICET, FIUBA, FADU, University of Buenos Aires, Pabellón III, subsuelo, Ciudad Universitaria, C1428EGA Buenos Aires, Argentina; 3 PhotoGreen Lab, Department of Chemistry, University of Pavia, Viale Taramelli 12, 27100 Pavia, Italy

**Keywords:** oxygen heterocycles, NMR spectroscopy, density functional calculations, Hammett correlation, Lynch correlation

## Abstract

The attribution of ^1^H and ^13^C NMR signals of a library of 5-, 6- and 7-substituted 2,2-dimethylchroman-4-one derivatives is reported. Substituent effects were interpreted in terms of the Hammett equation, showing a good correlation for carbons *para*- to the substituent group, not for the *meta*- ones. Similarly, the Lynch correlation shows the additivity of the substituent chemical shifts in the case of both H and C nuclei, again with the exception of the carbons in the *meta*- position. Density Functional Theory (DFT)-predicted ^1^H and ^13^C chemical shifts correspond closely with experimentally observed values, with some exceptions for C NMR data; however, the correlation is valid only for the aromatic moiety and cannot be extended to the heterocyclic ring of the chroman-4-one scaffold.

## 1. Introduction

Linear free-energy relationships (LFER) establish a direct correlation between thermodynamic parameters and kinetic data, offering insights into reaction mechanisms. LFERs create a connection between several measurable quantities and well-known parameters, Hammett substituent constants (either σ, σ^+^ or σ^−^) being perhaps the most renowned example. Some time ago, pioneering studies demonstrated that Hammett parameters can be correlated, not only with reaction rates and equilibrium constants, but also with ultraviolet absorption spectra, infrared frequencies, and NMR chemical shifts [1,2,3]. Indeed, recent studies showcased the importance of the Hammett linear correlation methodology in different fields of current interest [4,5,6,7]. For example, σ_P_ Hammett parameters were successfully correlated with the chemical shifts of ^1^H and ^13^C NMR spectra in solution of a series of tetrabutylammonium iron(III) porphyrin complexes, in turn revealing how the electronic effect of the substituents affected the electronic configuration [4]. Likewise, Hammett correlations were employed to analyze the variation of the strength of intramolecular H-bonds in substituted quinoline-3-carboxamide and 2-aryliminocoumarin-3-carboxamide derivatives. Nicely, the effect of the substituent depended on its conjugation with the proton-accepting group and, moreover, the effect was transferred via a through-space polarization [5]. Recently, Hammett correlation analyses have been performed to rationalize how geometric parameters, electronic structures, and the chemical reactivity of a library of pyrazole dyes were affected by the nature and type of attached substituents. Thus, good linear correlations were obtained when the energies of the HOMO and the LUMO were plotted against the substituent Hammett [6]. Very recently, an interesting work was reported, dealing with quantitative structure-property relationship (QSPR) models against NMR chemical shifts of a series of substituted phenylpyrimidine derivatives. Thus, Hammett parameters (σ, σ_F_ and σ_R_) were employed as a molecular descriptor to characterize the substituent effects [7].

The validity of such relationships comes from the definition of Hammett substituent constants themselves, which offer a measure of the relative effect (e.g., inductive and/or mesomeric) of a given substituent on the electron density at the portion of molecule under study. Accordingly, there should be a direct correlation with any property affected by the electron density at the considered site. Therefore, the rationalization of substituent effects propagation in a molecule leads to a deep understanding of structure-property relationships and shines light on reaction mechanisms, two essential concepts in chemistry [8,9,10].

Frequently, NMR spectroscopic parameters are used to understand the transmission of substituents electronic effects within organic molecules, with a specific focus on routinely used ^1^H and ^13^C chemical shift values [11,12,13]. Thus, Equation (1) describes the Hammett LFER correlating experimental C-NMR chemical shifts (δ(C–X)) with Hammett substituent constants (σ), where ρ is a constant reflecting the sensitivity of the chemical shift values to the substituent and δ_0_ is the chemical shift value of the unsubstituted compound [1].

δ(C–X) = ρ···σ + δ_0_ (1)

When good linear correlations are obtained applying Equation (1), the substituent effect on the chemical shift values can be considered electronic in origin, and the electron density around the nucleus under study is mostly affected by the electron-donating or -withdrawing ability of the substituent itself.

In recent years, computational chemistry, in particular Density Functional Theory (DFT), has provided strong support to experimental chemists, giving insight into the properties and reactivity of different molecular entities. This is particularly apparent in the realm of organic chemistry, where the behavior of a given molecular group (e.g., an aromatic nucleus) can be tuned by the nature of the attached substituents, if any. On one hand, it has been demonstrated that DFT can reproduce nicely the experimental data and, on the other one, it may have a predictive value as well, e.g., in those cases where experimental data are not available [14,15,16,17].

Herein, we report the application of these methods to 2,2-dimethylchroman-4-one derivatives, for which substituent effects have not been previously assessed. The chroman-4-one (2,3-dihydro-4-oxo-4H-1-benzopyran) ring system occupies an important position among oxygen heterocycles, as it is featured in a wide variety of compounds endowed with biological, medicinal, and synthetic interest [18,19,20,21,22,23,24]. The chroman-4-one parent system has been identified in several natural products [25,26,27] and marine organisms with therapeutic value [28]. Therefore, the vast range of biological effects associated with this scaffold has resulted in the chromanone ring system being considered a privileged structure [29].

**Scheme 1:**
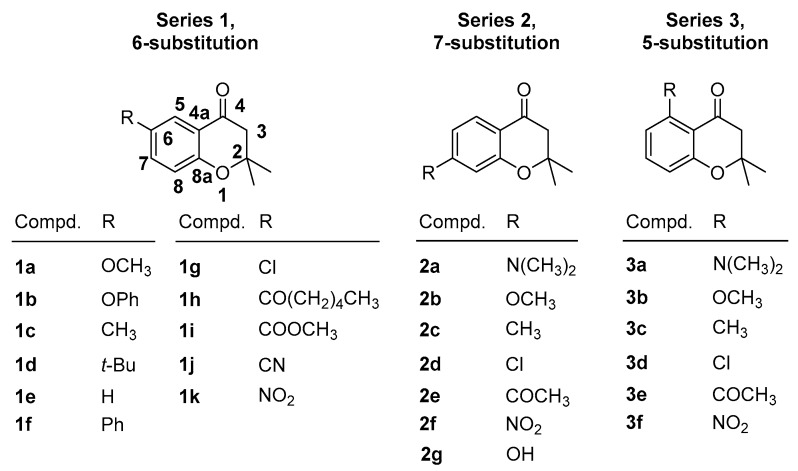
Structures of substituted 2,2-dimethylchroman-4-one derivatives studied in this work.

With the objective of understanding the factors underlying the transmission of substituents effect within the chromanone system, a library of substituted 2,2-dimethylchroman-4-one derivatives (Series 1–3, respectively) has been studied and their chemical structures are shown in Scheme 1, along with the numbering of the overall scaffold.

## 2. Results and Discussion

Based on our previous work and after an accurate survey of the literature, we decided to collect the ^1^H and ^13^C NMR spectroscopic data of 2,2-dimethylchroman-4-one derivatives from Series 1–3 [30,31,32]. In turn, these values have been exploited to analyze the effect connected with the introduction of a given substituent onto the parent structure in terms of: (i) *Lynch correlations* to evaluate the proportionality relationships with respect to the appropriate substituent chemical shift (SCS) in monosubstituted benzene; and (ii) *Hammett correlations* to show the resonance and inductive transmission of substituent electronic effects. Finally, prediction of the ^1^H and ^13^C nuclei chemical shifts by using DFT calculations has been also undertaken, followed by an analysis of the existing correlation among theoretical and experimental data.

### 2.1. Experimental NMR Chemical Shifts

The experimental ^1^H chemical shifts of chroman-4-one derivatives belonging to Series 1, 2 and 3 (see Scheme 1) are reported in Tables S1, S2 and S3, respectively (see Supplementary Materials —SM—). The substituent effect on the ^1^H chemical shift of the methylene group (CH_2_ at 3-position of the chroman-4-one moiety) displays a deshielding effect for all the studied compounds, as can be inferred from the data collected in the tables. Thus, this signal is located at *ca*. 2.70 ppm in the ^1^H-NMR spectra, as a result of the effect of both the carbonyl and the alkoxy groups belonging to the chroman-4-one scaffold. However, the substituents attached to the phenyl moiety (R in Scheme 1) do not pass on any substituent effect. Likewise, no effect is observed on the chemical shifts of the methyl groups (see 2-position) and their signals are located at *ca*. 1.40–1.50 ppm in the ^1^H-NMR spectra. As for the aromatic ring, the coupling constants (*J*) of the 6-substituted derivatives (Series 1) are those expected for this substitution pattern, with values of *J*_5,7_ from 1.6 to 2.4 Hz and *J*_7,8_ from 8.0 to 8.6 Hz. The *J* constants of compounds belonging to Series 2 (substituted in the 7-position) show values of *J*_5,6_ from 8.0 to 8.8 Hz and *J*_6,8_ from 1.0 to 2.4 Hz, while those of Series 3 (substituted in the 5-position) show values of *J*_6,7_ from 8.0 to 8.8 Hz and *J*_6,8_ from 1.0 to 2.4 Hz.

The ^13^C chemical shifts of the 2,2-dimethylchroman-4-one derivatives from Series 1, 2 and 3 are reported in Tables S4, S5 and S6, respectively (see SM). No substituent effect on the ^13^C chemical shifts of the methylene group in the 3-position and the methyl groups in 2-position were observed, as apparent from the data collected in the tables. A “*normal*” substituent effect on the ^13^C chemical shift of the carbonyl group was found for the compounds in Series 2, as can be judged from the deshielding effect on moving from electron-releasing to electron-withdrawing substituents, *viz.* 190.9 ppm for -NMe_2_ group vs. 195.0 and 201.3 ppm for -NO_2_ and -Cl substituents, respectively. On the other hand, no substituent effect on the same group was observed for derivatives from Series 1 and 3, due to a noticeable spread of the data. In addition, the substituent effect is nicely observed on *ortho*- and *para*- carbons for all the 2,2-dimethylchroman-4-one derivatives studied, while the same effect on the *meta*- carbons showed no linear trends due to the spread of the data (see below).

#### 2.1.1. Lynch Correlation

**Figure 1:**
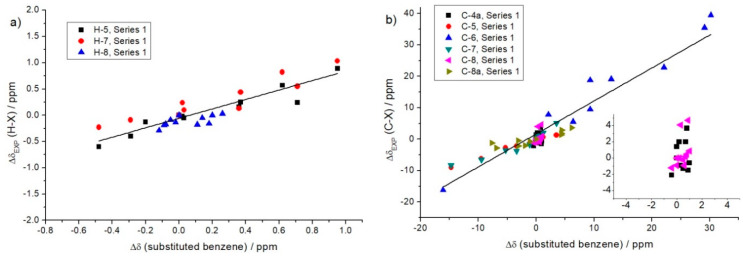
Lynch correlations of 2,2-dimethylchroman-4-one derivatives belonging to Series 1: (**a**) ^1^H and (**b**) ^13^C correlations; the inset in (**b**) shows the profile of carbons *meta*- to the substituent R (C-4a and C-8).

Analysis of substituents effect on the ^1^H and ^13^C chemical shifts of the investigated 2,2-dimethylchroman-4-ones according to the Lynch correlation was carried out [33]. This analysis gives an estimate of the substituent effect transmission on the considered scaffold (2,2-dimethylchroman-4-one) compared to the case of monosubstituted benzene. The Lynch correlation was constructed according to the following linear relationship (Equation (2)):

Δδ_EXP_ = **a**···Δδ(Benzene) + **b** (2)

where Δδ_EXP_ is the substituent chemical shifts (SCS) of ^1^H or ^13^C nuclei observed in 2,2-dimethylchroman-4-one derivatives, while Δδ(Benzene) is the SCS of ^1^H or ^13^C nuclei observed in monosubstituted benzene; **a** is the slope that compares the substituent effect on both aromatic moieties and **b** is the intercept. As an example, Figure 1a shows the results for the ^1^H nuclei of chroman-4-one derivatives belonging to Series 1, while the same analysis for the ^13^C nuclei of the aromatic ring is shown in Figure 1b. Furthermore, similar trends were consistently obtained for the compounds in Series 2 and 3 as well (see Figures S1 and S2, respectively, in the SM).

The obtained Lynch correlations are collected in Table 1 and it is apparent that the behavior depends on the considered molecules set and the NMR data. As for H-NMR, in all cases a good linear dependence was found (R-square = 0.8 for Series 1, > 0.9 for both Series 2 and 3), with a slope < 1 and an intercept around 0. Thus, Series 1 has the lowest slope value (0.90), while Series 2 and 3 consistently gave a slope around 0.95. Accordingly, we can conclude that the substituent effect on the hydrogen atoms attached to the benzene ring of the 2,2-dimethylchroman-4-one moiety is transmitted to a lower extent (**a** values < 1) than in the monosubstituted benzene ring and this is particularly apparent for compounds belonging to Series 1.

On the other hand, application of the Lynch equation to ^13^C NMR data depicts a different scenario, since the correlation cannot be extended to all the carbon atoms. In particular, the carbon atoms *meta*- with respect to the substituent R show a spread along the vertical axis for all the series and, accordingly, were not further considered in the data set (see inset in Figure 1b). Upon removal of such carbon atoms, good linear correlations were found, with R-square values > 0.90 (Table 1). Indeed, both Series 1 and 3 gave a slope slightly > 1 (around 1.05), while Series 2 had a slope of 0.94.

Notably, cumulative Lynch correlations of ^1^H and ^13^C nuclei for all the series (see Figure S3 in SM) consistently gave the expected linear trends. The linear correlation for ^1^H nuclei chemical shift can be represented by: Δδ_EXP_ = 0.91···Δδ(Benzene) − 0.04, with a R-square value of 0.90, while for ^13^C nuclei chemical shifts (carbons *meta*- to the substituent R were not considered), the Lynch correlation afforded the following relationship: Δδexp = 1.0 Δδ(Benzene) − 0.1, with a R-square value of 0.90. These results reinforce the conclusions for each series previously described.

#### 2.1.2. Hammett Correlation

**Figure 2:**
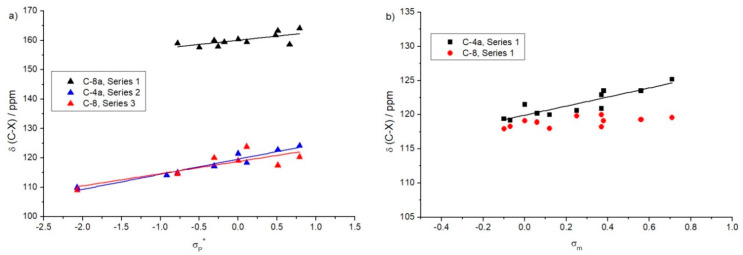
Hammett correlations between the ^13^C chemical shift values (δ(C-X)) of substituted 2,2-dimethylchroman-4-one derivatives and Hammett substituent constants (σ_p_^+^ or σ_m_, depending on the considered C-atoms): (**a**) carbon atoms in *para*- position (Series 1–3), and (**b**) carbon atoms in *meta*- position (Series 1) with respect to the substituent R.

Linear Hammett correlations for 2,2-dimethylchroman-4-one derivatives have been also investigated, adopting solely ^13^C chemical shifts as the probe to analyze this type of LFER. This choice was based on the advantages of carbon atoms over their hydrogen counterparts because ^13^C nuclei span over a larger chemical shift range and smaller dependence on medium effects is commonly observed. Thus, looking for a correlation involving ^13^C chemical shifts of chroman-4-one derivatives grouped in Series 1–3, different Hammett parameters have been screened, including σ_p_^+^, σ_m_ and σ_I_ values [3]. Figure 2a shows the linear correlation fittings that were obtained with the σ_p_^+^ Hammett parameters, when carbon atoms in the *para*- position with respect to the substituent R (C-8a for Series 1, C-4a for Series 2, and C-8 for Series 3) were considered. The choice of the σ_p_^+^ Hammett parameter is related to the specific position of the analyzed carbons, as well as to the presence in the chromanone scaffold of both the carbonyl and alkoxy groups attached at positions C-4a and C-8a, respectively. Accordingly, the attached R substituent can interact directly with the alkoxy or carbonyl groups through resonance effect [34,35,36]. Table 2 shows the Hammett correlations obtained by fitting the data in Figure 2a according to Equation (1). Thus, it is apparent that positive ρ values are obtained for all the series, indicating that the chemical shifts are sensitive to the electronic effect of the substituent R attached to the aromatic ring. In particular, electron-donating groups cause upfield shifts due to increased shielding of the nucleus, while electron-withdrawing analogs cause downfield shifts. It is worth noting that analogous linear Hammett correlations have been previously observed on 6- and 7-substituted coumarin derivatives, where the Hammett analyses have been consistently performed with σ_p_^+^ Hammett parameter [36,37]. Notably, at variance from Series 1 (δ_0_ around 160 ppm) Series 2 and 3 show very similar δ_0_ values (around 120 ppm), as a consequence of the different arrangement of the R substituent with respect to the carbonyl and the alkoxy groups of the chroman-4-one skeleton.

Next, we analyzed Hammett correlations of ^13^C chemical shifts of carbon atoms in the *meta*- position (see Table 2), by choosing σ_m_ and σ_I_ parameters as variables to account for the pure inductive effect of the substituents [3]. Thus, Figure 2b depicts as an example the correlations with the σ_m_ parameter for the two carbon atoms in *meta*- position with respect to the substituent R in Series 1 (all the other correlations can be found in Figures S4 and S5 in the SM). The data in Table 2 for compounds in Series 1 show that positive ρ values are found for C-4a, of a similar order of magnitude for both Hammett parameters (σ_m_ and σ_I_), indicating that the chemical shifts are sensitive to the inductive effect of the substituent attached to the aromatic ring. A similar behavior was observed for the C-8a of compounds in Series 2 with both Hammett parameters. On the other hand, no correlation (R-square consistently < 0.4) with either σ_m_ or σ_I_ Hammett parameters was observed for C-8 of Series 1, C-5 of Series 2 and for both C-7 and C-8a of Series 3. This behavior can be attributed to the fact that the electronic effect of the substituent is poorly transmitted to *meta*-carbons, resulting in modest chemical shift changes (<3 ppm range). Accordingly, subtle non-electronic effects on the chemical shift may have a role, preventing to obtain a correlation with Hammett parameters.

#### 2.1.3. Experimental and Theoretical (DFT Calculations) Correlation

**Figure 3:**
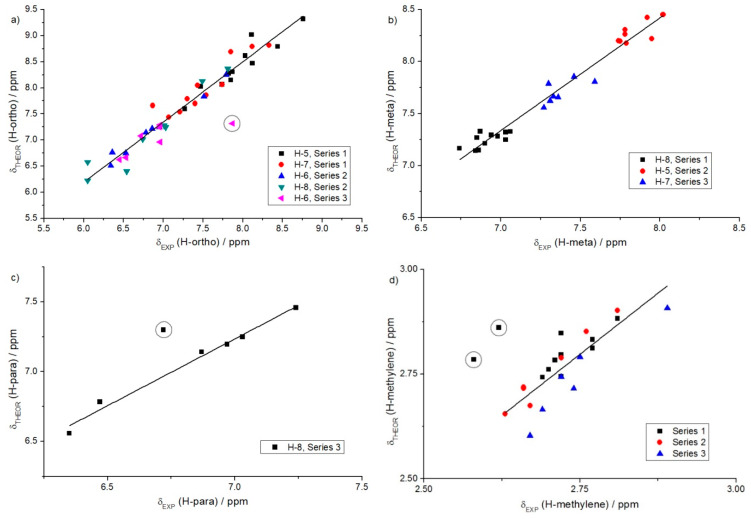
Correlation between experimental (δ_EXP_) and theoretical DFT (δ_THEOR_) chemical shifts for: (**a**) H-*ortho*; (**b**) H-*meta*; (**c**) H-*para* with respect to the substituent R group and (**d**) methylene CH_2_ moiety in substituted-2,2-dimethylchroman-4-ones of Series 1, 2 and 3; the data in the gray circles have not been considered for building the linear correlations (see text for details).

The results of DFT calculations for all the 2,2-dimethylchroman-4-one derivatives belonging to Series 1, 2, and 3 in terms of predicted ^1^H and ^13^C chemical shift values have been collected in Tables S7–S12 (see SM). The structures have been optimized by having recourse to the standard B3LYP functional with the 6-31G(d) basis set in the gas phase and the chemical shifts have been calculated via the “NMR” keyword at the B3LYP/6-311+G(2d,p) level of theory in chloroform bulk (see Materials and Methods section below for more details). Next, we have attempted to build a correlation between the theoretical ^1^H chemical shift values (δ_THEOR_) and the experimental ones (δ_EXP_), the corresponding plots being shown in Figure 3.

Independently from the relative arrangement (*ortho*-, *meta*- or *para*-) with respect to the substituent group R, nice linear correlations between the predicted and experimental H-NMR chemical shifts have been obtained with slopes near unity (between 0.96 and 1.16) and R-square ≥ 0.95 (see Table 3).

These results demonstrate that the prediction of the chemical shifts of the aromatic hydrogen atoms using the adopted theoretical approach is accurate, in turn leading to a good estimation of H-NMR chemical shifts. Different comments can be offered; however, depending on the considered data set. As for *ortho*-hydrogens (Figure 3a), their chemical shifts span a quite wide range, namely from around 6.0 up to almost 9.0, and the points are quite evenly distributed across the whole range, independently from the considered Series. One exception is represented by the case of compound **3f**, showing an important deviation from the fitting curve (see data point indicated by the gray circle in Figure 3a). The observed behavior was attributed to some specific interaction between the -NO_2_ moiety and the adjacent hydrogen atom (H-6). Thus, the nitro group is markedly tilted with respect to the aromatic plane in the modeled conformer of **3f** (see Figure S6 in SM), possibly causing the observed anomaly. Turning to *meta*-hydrogens (Figure 3b), all the data concur to build a single fitting curve with an overall nice correlation, albeit it is interesting to note that the data for each series accumulate in a different zone of the chart, with increasing chemical shift according to the relation: Series 1 < Series 3 < Series 2. This behavior can be explained considering the relative positions of the electron-withdrawing carbonyl group and the electron-donating alkoxy group of the chroman-4-one skeleton together with the substituents R. Thus, when moving from Series 1 (H-8 positioned *meta*- to carbonyl and R groups, and *ortho*- to the alkoxy one), to Series 3 (H-7 positioned *para*- to carbonyl, and *meta*- to alkoxy and R groups) and, finally, to Series 2 (H-5 positioned *ortho*- to carbonyl, and *meta*- to alkoxy and R groups), increasingly higher chemical shifts values can be expected. Moving to *para*-hydrogens (Figure 3c), a single Series contributes to this trend, namely Series 3, and it is again possible to observe a linear correlation, with the only exception of the point belonging to compound **3e** (see gray circle in Figure 3c), being excluded from the linear regression. Indeed, this anomalous behavior can be again related to the peculiar (non-planar) arrangement of the acetyl group in the considered absolute minimum conformation (see again Figure S6 in SM).

**Figure 4:**
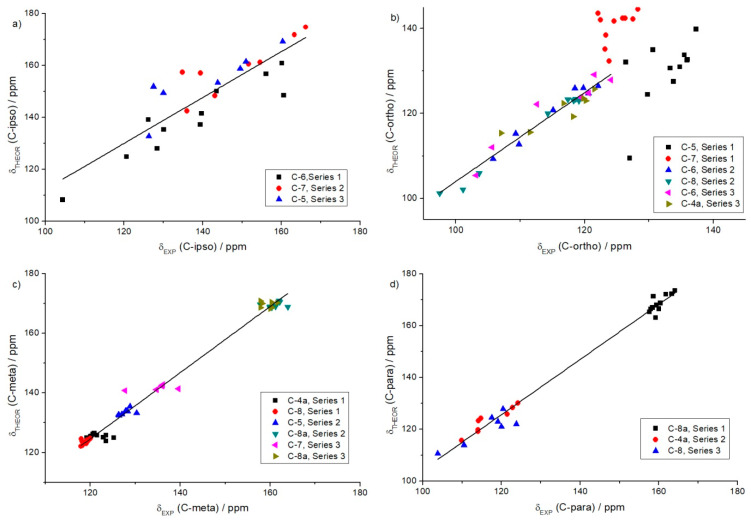
Correlation between experimental (δ_EXP_) and theoretical DFT (δ_THEOR_) chemical shifts for: (**a**) C-*ipso*; (**b**) C-*ortho*; (**c**) C-*meta* and (**d**) C-*para* with respect to the substituent R group in substituted-2,2-dimethylchroman-4-ones of Series 1, 2 and 3.

On the other hand, methylene groups of chroman-4-one derivatives give a worse linear correlation (Figure 4d) than the hydrogens from the aromatic ring, as hinted from the R-square value (*ca.* 0.71) reported in Table 3. Indeed, this correlation has been obtained by omitting from the fitting the data points related to derivatives **1e** and **1j**, which clearly deviate from the set of the other chroman-4-ones, albeit no specific geometric features can be related to such anomalous behavior. Furthermore, it is important to point out that the reported data have been calculated by considering the average chemical shift of the non-equivalent hydrogens (see the axial vs. equatorial arrangement), as obtained from the calculation carried out on the most stable conformer of each compound.

Taking into account that DFT calculations can predict successfully the chemical shifts of hydrogen nucleus, we also attempted to build a correlation between the predicted and experimental ^13^C chemical shifts, and the plots are shown in Figure 4. It is apparent that linear relationships were consistently obtained for carbon *ipso*-, *meta*-, and *para*- with respect to the substituent R for all the examined Series, with some exceptions in the case of *ortho*-carbons. The R-square values of the linear correlations are shown in Table 3. However, these results deserve some comments. Figure 4a shows a linear trend between the theoretical and experimental chemical shifts of *ipso*-carbons in Series 1–3 and the obtained slope value (0.88) is lower than unity. Indeed, the presence of a certain spread of data was found, resulting in a quite poor linear correlation (R-square = 0.75). On contrast, good linear correlations were obtained for the *ortho*-carbons in Series 2 and 3 (not in Series 1), as well as *meta*- and *para*-carbons in Series 1–3 (see Figure 4b–d) with slopes slightly higher than unity (1.04, 1.11 and 1.07, respectively), implying that the ^13^C NMR chemical shifts calculated by means of the DFT methodology predict with significant accuracy those obtained experimentally.

It is worth noting, however, that in the case of *meta*- and *para*-carbons, the considered data group together in different parts of the graph, leading to the observed correlation, while a quite flat distribution can be observed when considering the single sets of *meta*-carbons (see Figure 4c). Finally, a poor correlation was observed for the *ortho*- carbon atoms belonging to Series 1 (C-5 and C-7; Figure 4b). We have also attempted to build a correlation between the predicted and experimental ^13^C NMR chemical shifts of C-2, C-3 and the carbonyl group (C=O) of Series 1–3 and the plots are given in Figure S7 (see SM). However, no correlation was observed for these carbon atoms due to a significant spread of the data.

## 3. Materials and Methods

### 3.1. Spectroscopic Details

The synthesis and full characterization of the 2,2-dimethylchroman-4-one derivatives belonging to Series 1–3 have been reported in previous studies [30,31,32].

### 3.2. Computational Details

DFT calculations were carried out with the Gaussian09 (rev. D.01) software (Gaussian, Inc., Wallingford, CT, USA) [38]. All the 2,2-dimethylchroman-4-one derivatives have been initially optimized with recourse to the standard B3LYP functional and the 6-31G(d) basis set in the gas phase. To confirm that the optimized structures were minima, vibrational frequencies have been calculated at the same level of theory as geometry optimizations, and it was verified that they had only real frequencies. For each derivative, a systematic investigation on all of the possible conformations has been carried out. However, only the most stable geometry (absolute minimum) has been reported in the SM and considered for further work. NMR chemical shifts have been calculated via the “NMR” keyword with the Gauge-Independent Atomic Orbital (GIAO) method at the B3LYP/6-311+G(2d,p) level of theory in chloroform (the default parameters for the solvent effect have been adopted) on the geometry previously optimized in the gas phase. Thus, the theoretical chemical shifts reported in Tables S7–S12 have been calculated with the aid of the GaussView software from the GIAO nuclear magnetic shielding tensors obtained as calculations outputs by considering the data for TMS (tetramethylsilane, (CH_3_)_4_Si) calculated at the B3LYP/6-311+G(2d,p) level of theory as reference.

## 4. Conclusions

From the analysis of the ^1^H and ^13^C NMR Lynch correlations, an estimate of the substituent effect transmission on the nuclei of 2,2-dimethylchroman-4-ones compared to the case of monosubstituted benzenes can be inferred. The data reported for the first time in this work clearly reveal that in all cases good linear correlations were found. Indeed, ^1^H NMR Lynch correlations showed slope values of 0.90 (for Series 1), while Series 2 and 3 consistently gave a slope around 0.95. Likewise, application of Lynch equation to ^13^C NMR data showed good linear correlations and the slopes were found to be between 0.94 and 1.05, demonstrating again that the substituent effect is well transmitted, similarly to the case of monosubstituted benzenes. However, the carbons *meta*- to the substituent R showed no correlation.

On the other hand, good linear correlation fittings were observed between the σ_p_^+^ Hammett parameters and the carbon atoms in the *para*-position with respect to the substituent R belonging to Series 1–3 and, in all cases, the ρ values were positive indicating that the chemical shifts are sensitive to the electronic effects of the substituents attached to the aromatic ring. Indeed, the resonance effect of electron-donating groups causes upfield chemical shifts, while that of electron-withdrawing groups causes downfield chemical shifts. However, for the case of the ^13^C nuclei in *meta*- position with respect to the substituent R in Series 1–3, the correlation with σ_m_ and σ_I_ Hammett parameters demonstrated that these carbon atoms are not sufficiently sensitive to the substituent inductive effect. This behavior can be attributed to the fact that the chemical shift changes on *meta*- substitution are in every case of comparable magnitude (<3 ppm range), therefore subtle non-electronic effects on the chemical shift may have to be postulated.

Finally, nice linear dependences between the theoretically predicted and experimental hydrogen and carbon NMR chemical shifts were obtained with slopes near unity, with only a few exceptions in the case of C-NMR (notably for carbon atoms *ortho*- to the substituent). These results demonstrate that the prediction of the chemical shifts of the aromatic hydrogen and carbon atoms using the adopted theoretical approach is accurate, in turn leading to a good estimation of the desired values.

## Figures and Tables

**Table 1:** Lynch correlations of ^1^H and ^13^C chemical shifts of substituted 2,2-dimethylchroman-4-one derivatives.

**Lynch correlations of ^1^H chemical shifts: Δδ_EXP_ = a···Δδ(Benzene) + b**				
Compounds	a	b	R^2^	Hydrogen atoms
Series 1	0.90	−0.06	0.80	H-5; H-7; H-8
Series 2	0.95	−0.04	0.94	H-5; H-6; H-8
Series 3	0.94	0.00	0.98	H-6; H-7; H-8
**Lynch correlations of ^13^C chemical shifts: Δδ_EXP_ = a···Δδ(Benzene) + b**				
Compounds	a	b	R^2^	Carbon atoms
Series 1	1.05	1.7	0.92	C-5; C-6; C-7; C-8a
Series 2	0.94	−1.9	0.95	C-4a; C-6; C-7; C-8
Series 3	1.05	−0.1	0.90	C-4a; C-5; C-6; C-8

**a** and **b** are, respectively, the slope (that compares the substituent effect on 2,2-dimethylchroman-4-one vs. benzene derivatives) and the intercept of the correlation.

**Table 2:** Hammett correlations for ^13^C chemical shifts of substituted 2,2-dimethylchroman-4-one derivatives.

**Hammett correlations for ^13^C chemical shifts in *para*-positions (σ_p_^+^): δ(C-X) = ρ···σ_p_^+^ + δ_0_**				
Compounds	ρ	δ_0_	R^2^	Carbon atom
Series 1	2.8	160.0	0.41	C-8a
Series 2	5.1	119.6	0.93	C-4a
Series 3	4.1	118.8	0.62	C-8
**Hammett correlations for ^13^C chemical shifts in *meta*-positions (σ_m_): δ(C-X) = ρ···σ_m_ + δ_0_**				
Compounds	ρ	δ_0_	R^2^	Carbon atom
Series 1	6.6	119.9	0.77	C-4a
	No correlation			C-8
Series 2	No correlation			C-5
	4.7	160.1	0.45	C-8a
Series 3	No correlation			C-7
	No correlation			C-8a
**Hammett correlations for ^13^C chemical shifts in *meta*-positions (σ_I_): δ(C-X) = ρ···σ_I_ + δ_0_**				
Compounds	ρ	δ_0_	R^2^	Carbon atom
Series 1	5.8	120.0	0.49	C-4a
	No correlation			C-8
Series 2	No correlation			C-5
	5.6	159.6	0.40	C-8a
Series 3	No correlation			C-7
	No correlation			C-8a

**σ** is the considered Hammett substituent constant; **ρ** and **δ_0_** are, respectively, the slope (that reflects the sensitivity of the chemical shift values to the substituent) and the intercept (the chemical shift value of the unsubstituted compound) of the correlation.

**Table 3:** Linear correlation between DFT calculated and experimental ^1^H and ^13^C NMR chemical shifts of substituted 2,2-dimethylchroman-4-one derivatives.

δ_THEOR_ = **a**···δ_EXP_ + **b**				
**Hydrogen atoms type ^a^**	**a**	**b**	**R^2^**	**Comments**
Ortho	1.16	−0.74	0.95	Series 1 (H-5, H-7); Series 2 (H-6, H-8); Series 3 (H-6)
Meta	1.08	−0.23	0.96	Series 1 (H-8); Series 2 (H-5); Series 3 (H-7)
Para	0.96	0.54	0.98	Series 3 (H-8)
Methylene	1.17	−0.42	0.71	H-3 of all series
**Carbon atoms type**	**a**	**b**	**R^2^**	**Comments**
Ipso	0.88	23.93	0.75	Series 1 (C-6); Series 2 (C-7); Series 3 (C-5)
Ortho	1.04	−0.71	0.93	Series 2 (C-6, C-8); Series 3 (C-6, C-4a)
	No correlation			Series 1 (C-5)
	No correlation			Series 1 (C-7)
Meta	1.11	−8.78	0.99	Series 1 (C-4a, C-8); Series 2 (C-5, C-8a); Series 3 (C-7, C-8a)
Para	1.07	−2.25	0.99	Series 1 (C-8a); Series 2 (C-4a); Series 3 (C-8)

**a** and **b** are, respectively, the slope (that compares the substituent effect between theoretical vs. experimental chemical shifts) and the intercept of the correlation. ^a^ In all cases, the fitting procedures were performed by excluding the data reported in the gray circle in Figure 3a–d.

## Supplementary Materials

The following are available online, Figures S1–S3: Lynch correlations; Figures S4 and S5: Hammett correlations; Figure S6: Optimized structures; Figure S7: Theoretical vs. experimental data correlation; Tables S1–S12: Experimental and calculated chemical shifts; Chromanones optimized structures.
