# Supplementary material for: Substituent Effects on NMR Spectroscopy of 2,2-Dimethylchroman-4-one Derivatives: Experimental and Theoretical Studies
Source: Molecules. 2020 Apr 28;25(9):2061. doi: 10.3390/molecules25092061 (PMC7248910; doi:10.3390/molecules25092061)
Supplement: Supplementary file 1 [file molecules-25-02061-s001.pdf]

Substituent Effects on NMR Spectroscopy of 2,2-Dimethylchroman-4-one Derivatives: Experimental and Theoretical Studies

Daniela Iguchi [1,2], Davide Ravelli [3,*], Rosa Erra-Balsells [1] and Sergio M. Bonesi [1,3,*]

1 CIHIDECAR-CONICET – Departamento de Química Orgánica – Facultad de Ciencias Exactas y Naturales, 3er Piso, Pabellón 2, Ciudad Universitaria, CP 1428, University of Buenos Aires, Argentina.
2 Present address: Design and Chemistry of Macromolecules Group, Institute of Technology in Polymers and Nanotechnology (ITPN), UBA-CONICET, FIUBA, FADU, University of Buenos Aires, Pabellón III, subsuelo, Ciudad Universitaria (C1428EGA), Buenos Aires, Argentina.
3 PhotoGreen Lab, Department of Chemistry, University of Pavia, Viale Taramelli 12, 27100 Pavia, Italy.
* Correspondence: davide.ravelli@unipv.it (D.R.); smbonesi@qo.fcen.uba.ar (S.M.B.)

Supplementary Materials

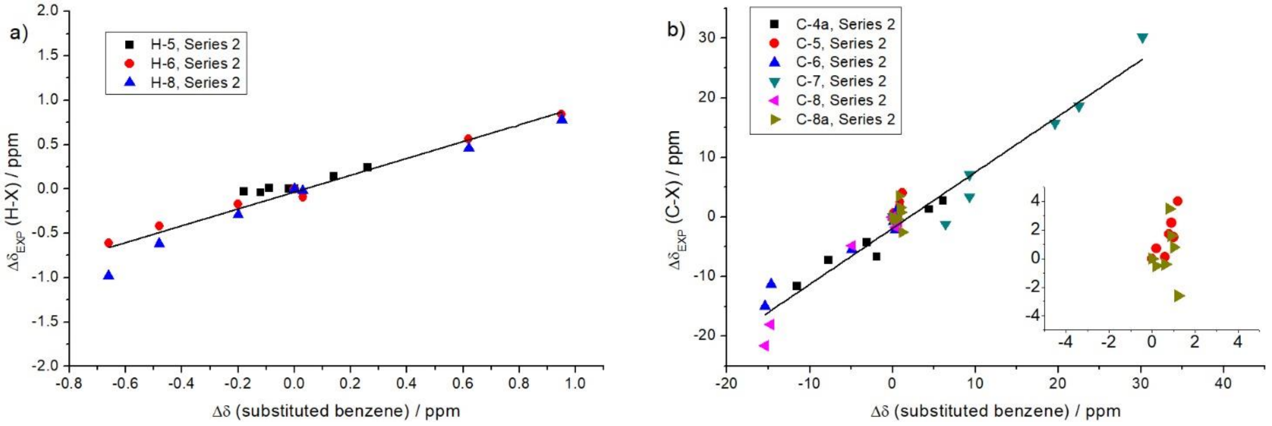

Figure S1. Lynch correlations of 2,2-dimethylchroman-4-one derivatives belonging to Series 2: (a) ^{1}H and (b) ^{13}C correlations; the inset in (b) shows the profile of carbons *meta*-to the substituent (C-5 and C-8a).

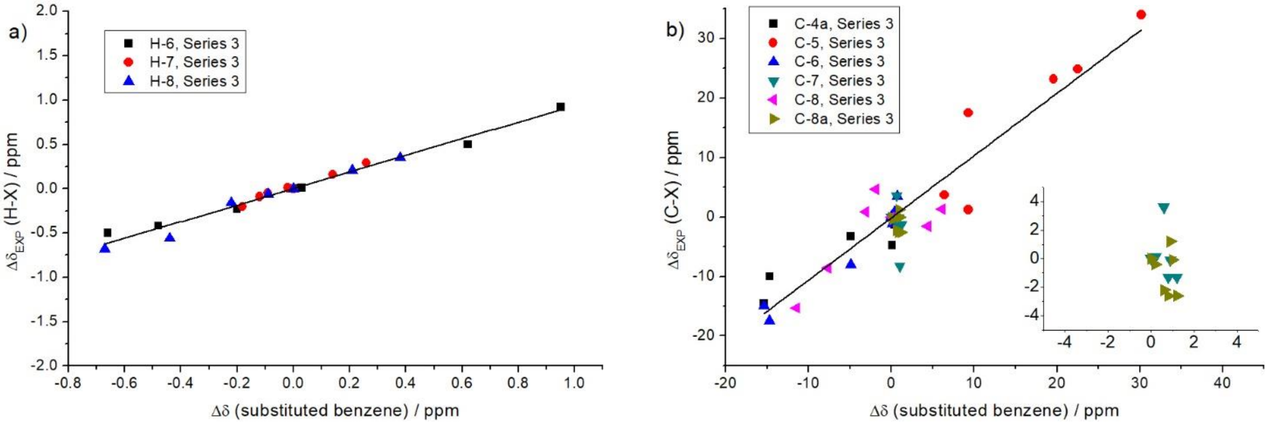

Figure S2. Lynch correlations of 2,2-dimethylchroman-4-one derivatives belonging to Series 3: (a) ^{1}H and (b) ^{13}C correlations; the inset in (b) shows the profile of carbons *meta*-to the substituent (C-7 and C-8a).

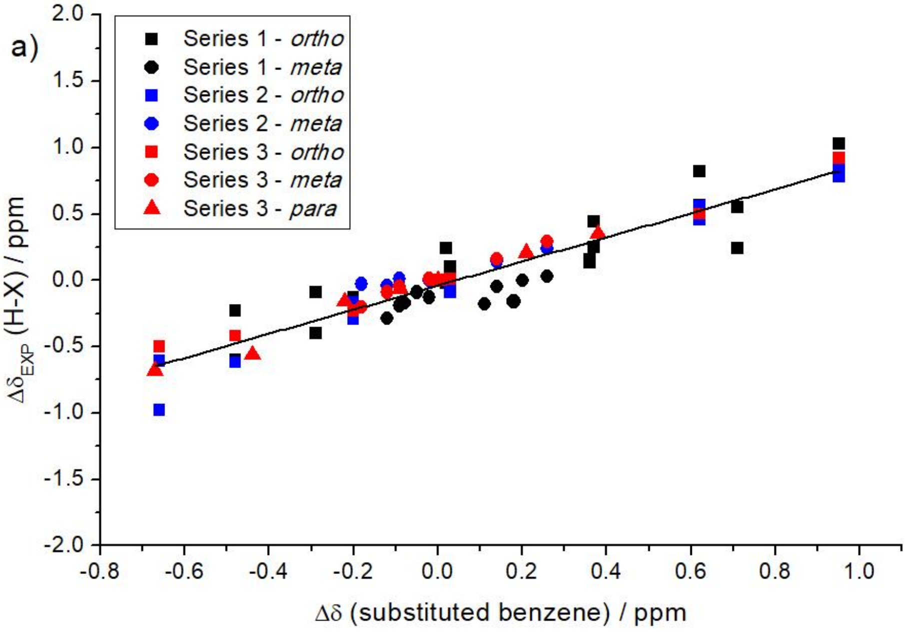

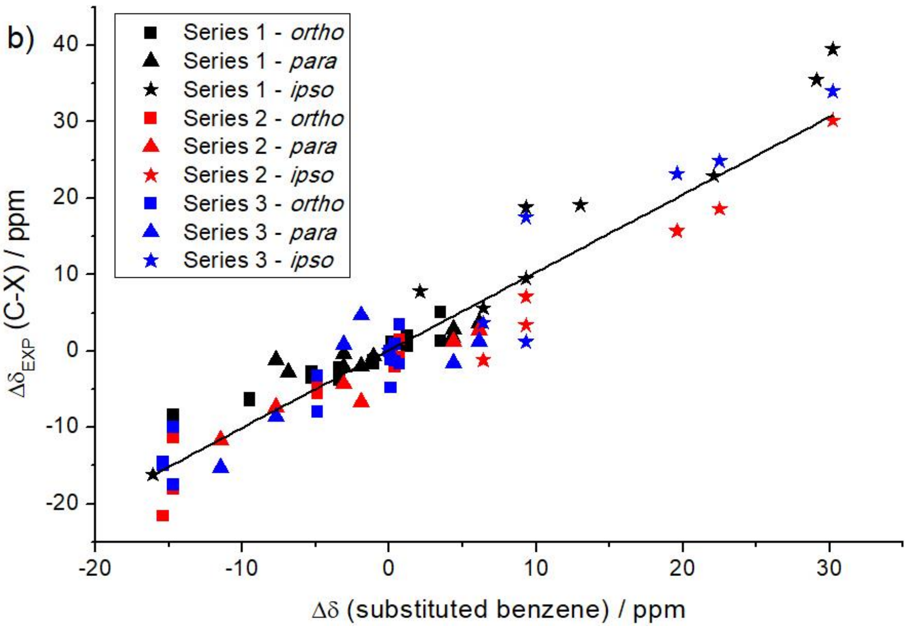

Figure S3. Lynch correlations of 2,2-dimethylchroman-4-one derivatives: (a) ^{1}H correlation of Series 1 (black symbols), Series 2 (blue symbols) and Series 3 (red symbols); (b) ^{13}C correlation of Series 1 (black symbols), Series 2 (blue symbols) and Series 3 (red symbols). For clarity reasons, ortho- (■); meta- (•), para- (▲) and ipso- (★) positions have been expressed in relation to the position of the substituent (R group).

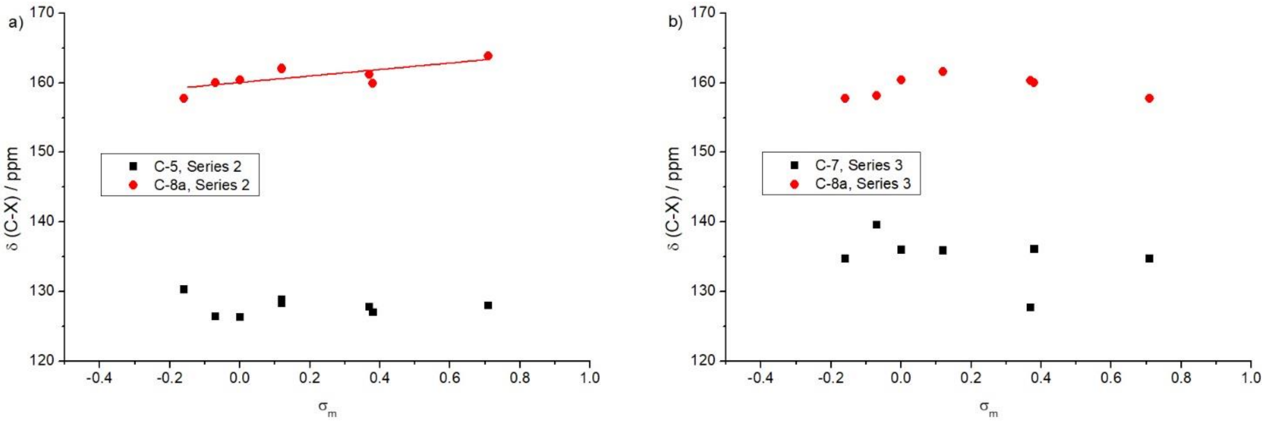

Figure S4. Hammett correlations between the ^{13}C chemical shift values (δ(C-X)) and σ_m Hammett substituent constants of substituted 2,2-dimethylchroman-4-one derivatives: carbon atoms in *meta*- position with respect to the substituent R for (a) Series 2 and (b) Series 3.

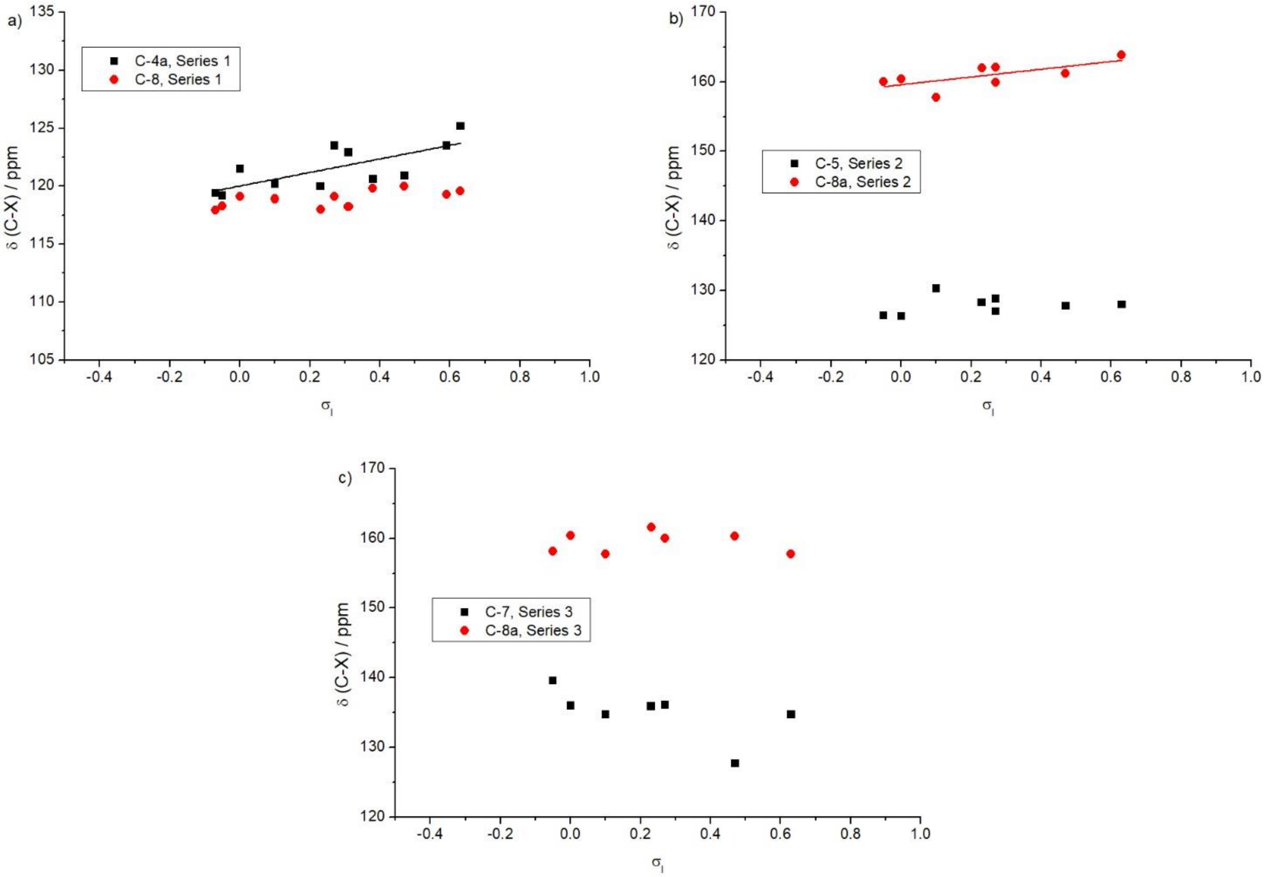

Figure S5. Hammett correlations between the ^{13}C chemical shift values (δ(C-X)) and σ_I Hammett substituent constants of substituted 2,2-dimethylchroman-4-one derivatives: carbon atoms in *meta*- position with respect to the substituent R for (a) Series 1, (b) Series 2 and (c) Series 3.

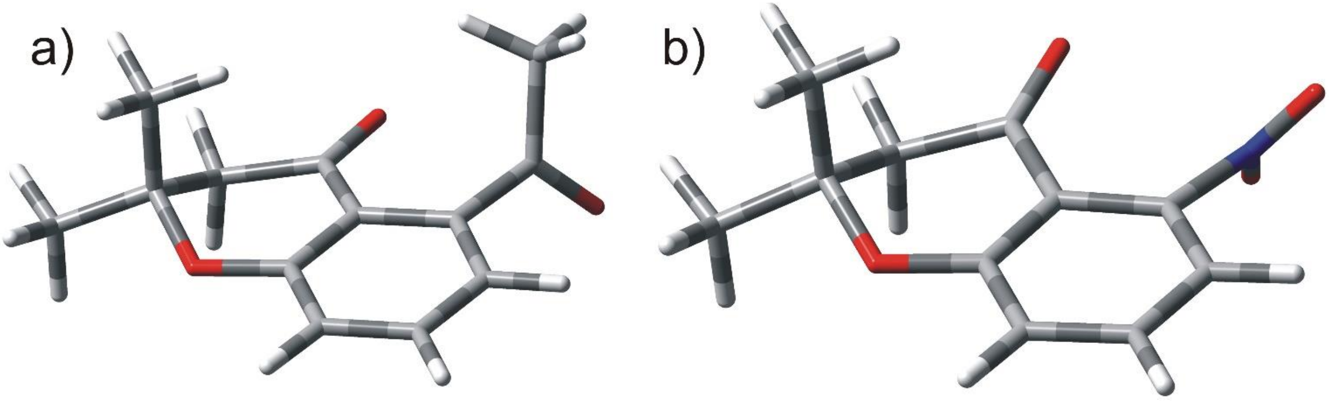

Figure S6. Optimized structures of 2,2-dimethylchroma-4-one derivatives: (a) **3e** and (b) **3f**.

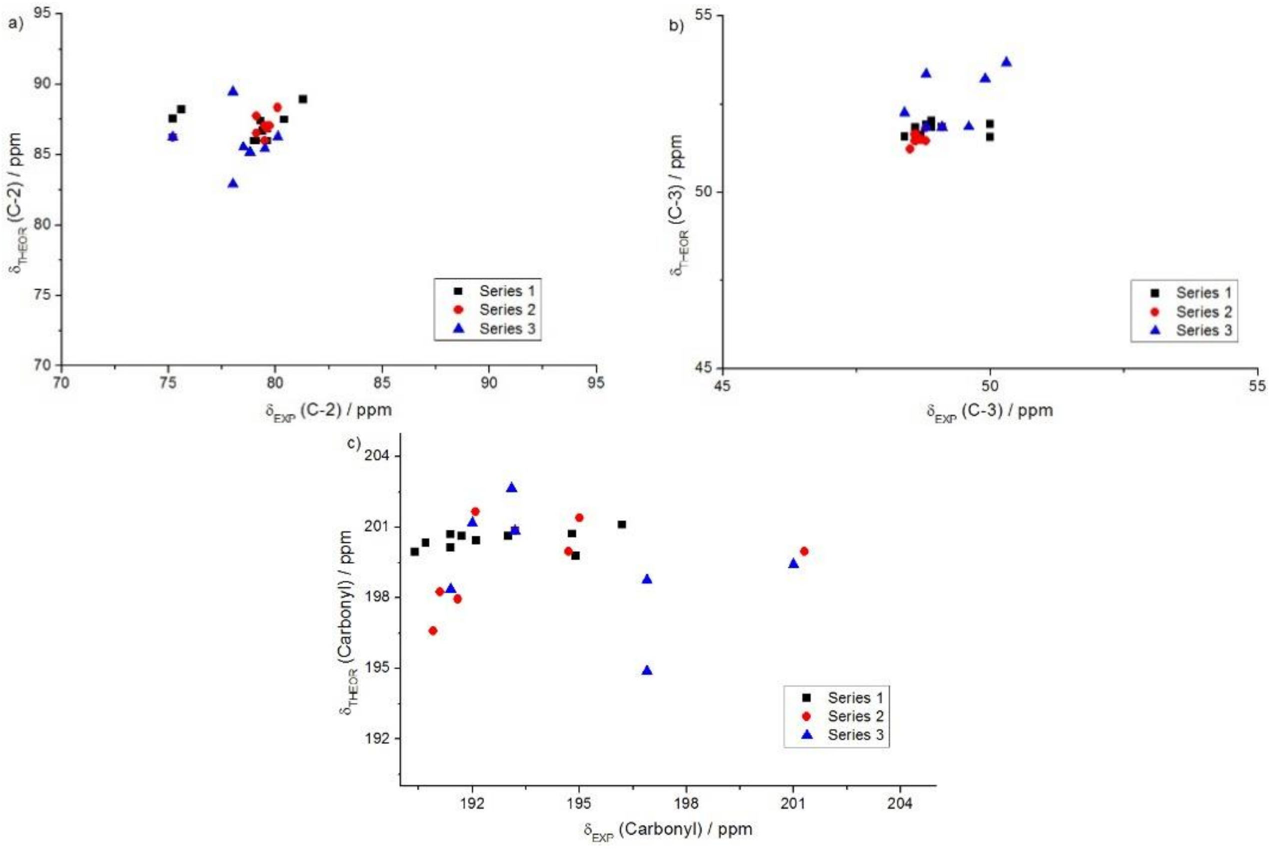

Figure S7. Correlation between experimental (δ_{EXP}) and theoretical DFT (δ_{THEOR}) chemical shifts for: (a) C-2, (b) C-3 and (c) carbonyl group (C=O) in substituted-2,2-dimethylchroman-4-ones of Series 1, 2 and 3.

Table S1. ^1H Chemical shifts of 6-substituted 2,2-dimethyl-4-chromanones (Series 1).[a]

Compound	R	H-5	H-6	H-7	H-8	Others
**1a**	OMe	7.27	---	7.07	6.84	3.82 (OCH_3); 2.70 (CH_2); 1.52 (CH_3)
**1b**	OPh	7.47	---	7.21	6.94	7.34 (4'-H); 7.09 (3'-H); 6.97 (2'-H); 2.72 (CH_2); 1.48 (CH_3)
**1c**	Me	7.74	---	6.87	6.74	2.69 (CH_2); 2.35 ($ArCH_3$); 1.45 (CH_3)
**1d**	*t*-Bu	7.85	---	7.54	6.86	2.71 (CH_2); 1.46 (CH_3); 1.31 (*t*-Bu)
**1e**	H	7.87	6.95	7.30	7.03	2.58 (CH_2); 1.51 (CH_3)
**1f**	Ph	8.12	---	7.74	7.03	7.59 (4'-H); 7.57-7.33 (3'-H and 2'-H); 2.77 (CH_2); 1.51 (CH_3)
**1g**	Cl	7.82	---	7.40	6.90	2.72 (CH_2); 1.46 (CH_3)
**1h**	CO*n*-Bu	8.44	---	8.12	6.98	2.95 (CH_2); 2.77 (CH_2); 1.70 (CH_2); 1.45 (CH_3); 1.42 (CH_2); 1.40 (CH_2); 0.94 (CH_3)
**1i**	COOMe	8.11	---	7.85	6.85	3.90 ($COOCH_3$); 2.72 (CH_2); 1.48 (CH_3)
**1j**	CN	8.03	---	7.43	6.87	2.62 (CH_2); 1.48 (CH_3)
**1k**	NO_2	8.76	---	8.33	7.06	2.81 (CH_2); 1.52 (CH_3)

[a] Values in parts per million (ppm). Internal reference: Tetramethylsilane.

Table S2. ^1H Chemical shifts of 7-substituted 2,2-dimethyl-4-chromanones (Series 2).[a]

Compound	R	H-5	H-6	H-7	H-8	Others
**2a**	NMe_2	7.75	6.34	---	6.05	2.90 ($N(CH_3)_2$); 2.63 (CH_2); 1.45 (CH_3)
**2b**	OMe	7.79	6.53	---	6.05	3.82 (OCH_3); 2.66 (CH_2); 1.45 (CH_3)
**2c**	Me	7.74	6.78	---	6.74	2.66 (CH_2); 2.35 ($ArCH_3$); 1.45 (CH_3)
**1e**	H	7.78	6.95	7.30	7.03	2.58 (CH_2); 1.51 (CH_3)
**2d**	Cl	7.78	6.86	---	7.01	2.72 (CH_2); 1.46 (CH_3)
**2e**	COMe	7.92	7.51	---	7.49	2.76 (CH_2); 2.60 ($COCH_3$); 1.47 (CH_3)
**2f**	NO_2	8.02	7.79	---	7.81	2.81 (CH_2); 1.52 (CH_3)
**2g**[b]	OH	7.95	6.36	---	6.54	2.67 (CH_2); 1.46 (CH_3)

[a] Values in parts per million (ppm). Internal reference: Tetramethylsilane. [b] Data taken from Ref. S1.

Table S3. ^{1}H Chemical shifts of 5-substituted 2,2-dimethyl-4-chromanones (Series 3).[a]

Compound	R	H-5	H-6	H-7	H-8	Others
**3a**	NMe_2	---	6.45	7.27	6.35	3.04 ($N(CH_3)_2$); 2.67 (CH_2); 1.46 (CH_3)
**3b**	OMe	---	6.53	7.36	6.47	3.91 (OMe); 2.69 (CH_2); 1.43 (CH_3)
**3c**	Me	---	6.72	7.33	6.87	2.72 (CH_2); 2.23 ($ArCH_3$); 1.47 (CH_3)
**1e**	H	7.78	6.95	7.30	7.03	2.58 (CH_2); 1.51 (CH_3)
**3d**	Cl	---	6.96	7.31	6.97	2.75 (CH_2); 1.46 (CH_3)
**3e**	COMe	---	6.96	7.46	6.72	2.74 (CH_2); 2.44 ($COCH_3$); 1.47 (CH_3)
**3f**	NO_2	---	7.87	7.59	7.24	2.89 (CH_2); 1.48 (CH_3)

[a] Values in parts per million (ppm). Internal reference: Tetramethylsilane.

Table S4. ^{13}C Chemical shifts of 6-substituted 2,2-dimethyl-4-chromanones (Series 1).[a]

Compound	R	C-5	C-6	C-7	C-8	C-8a	C-4a	Others
**1a**	OMe	127.0	160.1	123.8	118.0	159.2	120.0	194.8 (C=O); 79.6 (C-2); 54.3 (OCH_3); 48.8 (CH_2); 20.1 (CH_3)
**1b**	OPh	129.8	156.1	123.2	119.8	157.6	120.6	192.1 (C=O); 150.6 (1'-C); 128.2 (3'-C); 115.6 (2'-C); 79.3 (C-2); 48.7 (CH_2); 26.7 (CH_3)
**1c**	Me	126.4	139.4	122.1	118.3	160.0	119.2	191.4 (C=O); 79.1 (C-2); 48.8 (CH_2); 35.1 ($ArCH_3$); 26.7 (CH_3)
**1d**	*t*-Bu	133.8	143.5	122.5	117.9	157.9	119.4	193.0 (C=O); 79.0 (C-2); 48.9 (CH_2); 35.2 ($\underline{C}(CH_3)_3$); 31.4 ($C(\underline{C}H_3)_3$); 26.7 (CH_3)
**1e**	H	136.0	120.6	126.3	119.1	160.4	121.5	193.2 (C=O); 75.2 (C-2); 49.1 (CH_2); 24.9 (CH_3)
**1f**	Ph	134.8	139.7	124.6	118.9	159.4	120.2	196.2 (C=O); 133.8 (C-1'); 128.9 (4'-H); 127.2 (3'-C); 126.7 (2'-H); 79.4 (C-2); 48.9 (CH_2); 26.7 (CH_3)
**1g**	Cl	135.9	126.2	125.9	120.0	158.4	120.9	191.4 (C=O); 79.6 (C-2); 48.6 (CH_2); 26.5 (CH_3)
**1h**	CO*n*-Bu	135.5	130.1	127.5	119.1	163.3	123.5	198.7 and 191.7 (C=O); 80.4 (C-2); 48.6, 38.0 and 26.6 (CH_2); 22.5 and 14.0 (CH_3)
**1i**	COOMe	130.6	128.4	128.3	118.2	161.8	122.9	190.7 and 165.5 (C=O); 75.2 (C-2); 51.9 ($COO\underline{C}H_3$); 50.0 (CH_2); 26.4 and 24.2 (CH_3)
**1j**	CN	137.3	104.4	131.4	119.3	158.6	123.5	194.9 (C=O); 117.6 ($\underline{C}N$); 75.6 (C-2); 50.0 (CH_2); 26.4 (CH_3)
**1k**	NO_2	133.3	160.6	123.3	119.6	164.1	125.2	190.4 (C=O); 81.3 (C-2); 48.4 (CH_2); 26.6 (CH_3)

[a] Values in parts per million (ppm). Internal reference: Tetramethylsilane.

Table S5. ^{13}C Chemical shifts of 7-substituted 2,2-dimethyl-4-chromanones (Series 2).[a]

Compound	R	C-5	C-6	C-7	C-8	C-8a	C-4a	Others
**2a**	NMe_2	130.3	105.8	154.6	97.5	157.8	109.9	190.9 (C=O); 79.5 (C-2); 48.9 (CH_2); 40.3 ($N(CH_3)_2$); 26.8 (CH_3)
**2b**	OMe	128.3	109.3	166.2	101.1	162.0	114.1	191.1 (C=O); 79.6 (C-2); 55.6 (OMe); 48.6 (CH_2); 26.7 (CH_3)
**2c**	Me	126.4	122.1	139.4	118.3	160.0	114.2	194.7 (C=O); 79.1 (C-2); 48.8 (CH_2); 26.7 (CH_3); 21.9 ($ArCH_3$)
**2e**	H	126.3	120.6	136.0	119.1	160.4	121.5	193.2 (C=O); 75.2 (C-2); 49.1 (CH_2); 24.9 (CH_3)
**2d**	Cl	127.8	118.5	134.8	117.4	161.2	114.8	201.3 (C=O); 79.1 (C-2); 48.7 (CH_2); 26.5 (CH_3)
**2e**	COMe	127.0	119.8	143.1	118.6	159.9	122.8	197.4 and 192.1 (C=O); 79.7 (C-2); 48.8 (CH_2); 27.0 ($COCH_3$); 26.6 (CH_3)
**2f**	NO_2	128.0	115.1	151.7	114.3	163.9	124.2	195.0 (C=O); 80.1 (C-2); 48.6 (CH_2); 27.7 (CH_3)
**2g**[b]	OH	128.8	109.8	163.3	103.6	162.1	114.1	191.6 (C=O); 79.5 (C-2); 48.5 (CH_2); 26.6 (CH_3)

[a] Values in parts per million (ppm). Internal reference: Tetramethylsilane. [b] Data from Ref. S1.

Table S6. ^{13}C Chemical shifts of 5-substituted 2,2-dimethyl-4-chromanones (Series 3).[a]

Compound	R	C-5	C-6	C-7	C-8	C-8a	C-4a	Others
**3a**	NMe_2	151.0	105.6	134.7	103.8	157.8	107.0	196.9 (C=O); 78.0 (C-2); 40.3 ($N(CH_3)_2$); 49.6 (CH_2); 40.3 (CH_3)
**3b**	OMe	160.3	103.1	135.9	110.5	161.6	111.5	191.4 (C=O); 78.5 (C-2); 56.1 (OMe); 50.3 (CH_2); 26.4 (CH_3)
**3c**	Me	127.5	124.1	139.6	120.0	158.2	119.8	193.1 (C=O); 78.8 (C-2); 48.8 (CH_2); 22.0 ($ArCH_3$); 26.8 (CH_3)
**1e**	H	126.3	120.6	136.0	119.1	160.4	121.5	193.2 (C=O); 75.2 (C-2); 49.1 (CH_2); 24.9 (CH_3)
**3d**	Cl	130.0	121.5	127.7	123.8	160.3	120.2	201.0 (C=O); 80.1 (C-2); 49.9 (CH_2); 26.6 (CH_3)
**3e**	COMe	143.8	119.5	136.1	117.5	160.0	116.7	205.1 and 192.0 (C=O); 79.5 (C-2); 48.8 (CH_2); 30.8 and 26.5 (CH_3)
**3f**	NO_2	149.5	112.6	134.7	120.4	157.8	118.2	196.9 (C=O); 78.0 (C-2); 48.4 (CH_2); 26.8 (CH_3)

[a] Values in parts per million (ppm). Internal reference: Tetramethylsilane.

Table S7. Calculated [1]H Chemical shifts of the hydrogen atoms in 6-substituted 2,2-dimethyl-4-chromanones (Series 1).[a]

Compound	R	H-5	H-6	H-7	H-8	H-3	
**1a**	OMe	7.5971	--	7.4396	7.1436	2.5728	2.9489
**1b**	OPh	8.0260	--	7.5393	7.2948	2.5063	2.9817
**1c**	Me	8.0660	--	7.6594	7.1668	2.5398	2.9450
**1d**	*t*-Bu	8.1514	--	7.8624	7.1479	2.6115	2.9547
**1e**	H	8.3068	7.2485	7.7876	7.2485	2.5838	2.9853
**1f**	Ph	8.4710	--	8.0600	7.3224	2.5940	3.0293
**1g**	Cl	8.2803	--	7.6990	7.2125	2.6345	2.9573
**1h**	CO*n*-Bu	8.7931	--	8.7931	7.2828	2.6579	3.0073
**1i**	COOMe	9.0175	--	8.6897	7.2693	2.6874	3.0085
**1j**	CN	8.6152	--	8.0441	7.3300	2.7175	3.0045
**1k**	NO_2	9.3218	--	8.8126	7.3265	2.7551	3.0105

[a] Values in parts per million (ppm).

Table S8. Calculated [1]H Chemical shifts of the hydrogen atoms in 7-substituted 2,2-dimethyl-4-chromanones (Series 2).[a]

Compound	R	H-5	H-6	H-7	H-8	H-3
**2a**	NMe_2	8.1946	6.5088	--	6.2224	
**2b**	OMe	8.1773	6.7477	--	6.5789	
**2c**	Me	8.2005	7.1426	--	7.0152	
**1e**	H	8.3068	7.2485	7.7876	7.2485	
**2d**	Cl	8.2630	7.2192	--	7.2786	
**2e**	COMe	8.4232	7.8326	--	8.1261	
**2f**	NO_2	8.4518	8.2482	--	8.3662	
**2g**	OH	8.2186	6.7648	--	6.4006	

[a] Values in parts per million (ppm).

Table S9. Calculated ^{1}H Chemical shifts of the hydrogen atoms in 5-substituted 2,2-dimethyl-4-chromanones (Series 3).[a]

Compound	R	H-5	H-6	H-7	H-8	H-3
**3a**	NMe_2	--	6.6246	7.5573	6.5576	
**3b**	OMe	--	6.6650	7.6583	6.7839	
**3c**	Me	--	7.0808	7.6651	7.1407	
**1e**	H	8.3068	7.2485	7.7876	7.2485	
**3d**	Cl	--	7.2884	7.6235	7.1952	
**3e**	COMe	--	6.9634	7.8554	7.2992	
**3f**	NO_2	--	7.3180	7.8082	7.4574	

[a] Values in parts per million (ppm).

Table S10. ^{13}C Chemical shifts of the parent chromanone skeleton in 6-substituted 2,2-dimethyl-4-chromanones (Series 1).[a]

Compdound	R	C-2	C-3	C-4	C-4a	C-5	C-6	C-7	C-8	C-8a
**1a**	OMe	85.9948	51.9108	200.7273	125.3363	109.4700	160.8105	132.3402	124.4906	163.0373
**1b**	OPh	87.3941	51.6222	200.4575	126.1874	124.4514	156.7334	135.1037	124.5723	165.2650
**1c**	Me	85.9871	51.8598	200.6976	124.9005	132.0407	137.1892	143.5284	122.4533	166.5001
**1d**	*t*-Bu	85.9950	52.0323	200.6312	124.3274	127.5391	150.0318	141.9981	122.0753	166.4156
**1e**	H	86.2306	51.8539	200.8510	125.7788	132.6942	124.7722	142.4204	122.9802	168.7422
**1f**	Ph	86.6956	51.8446	201.1034	125.5267	130.9713	141.4573	141.6952	123.3137	167.9006
**1g**	Cl	86.8335	51.5791	200.1309	126.4171	132.5582	139.0757	142.4250	124.5990	166.9774
**1h**	CO*n*-Bu	87.4972	51.8319	200.6340	123.7977	133.7745	135.3131	142.2012	123.4580	172.2672
**1i**	COOMe	87.5672	51.9344	200.3257	125.0918	134.9622	128.0145	144.5112	123.6736	172.0924
**1j**	CN	88.1990	51.5680	199.7879	125.7553	139.8016	108.2498	146.4082	124.1161	171.3569
**1k**	NO_2	88.9235	51.5889	199.9510	124.9436	130.6739	148.4857	138.4123	123.8969	173.5838

[a] Values in parts per million (ppm).

Table S11. ^{13}C Chemical shifts of the parent chromanone skeleton in 7-substituted 2,2-dimethyl-4-chromanones (Series 2).[a]

Compdound	R	C-2	C-3	C-4	C-4a	C-5	C-6	C-7	C-8	C-8a
**2a**	NMe_2	86.0085	51.5234	196.6070	115.6618	133.2016	109.3177	161.1878	101.2225	169.6254
**2b**	OMe	86.8785	51.4589	198.2447	119.0816	133.7344	115.2590	174.7280	102.0735	170.9073
**2c**	Me	86.5465	51.4545	199.9601	123.2087	132.4305	126.4716	157.0828	123.0549	168.9930
**1e**	H	86.2306	51.8539	200.8510	125.7788	132.6942	124.7722	142.4204	122.9802	168.7422
**2d**	Cl	87.7302	51.4973	199.9817	124.2095	133.8723	125.9185	157.4176	123.3365	169.0499
**2e**	$COCH_3$	87.0739	51.8349	201.6456	128.3991	132.8061	126.0167	148.2755	123.4337	168.7350
**2f**	NO_2	88.3531	51.6561	201.3847	130.1573	133.8568	120.8088	160.4970	119.9712	168.8260
**2g**	OH	87.0639	51.2222	197.9475	119.8401	135.4443	112.7251	171.7694	105.9036	170.5189

[a] Values in parts per million (ppm).

Table S12. ^{13}C Chemical shifts of the parent chromanone skeleton in 5-substituted 2,2-dimethyl-4-chromanones (Series 3).[a]

Compdound	R	C-2	C-3	C-4	C-4a	C-5	C-6	C-7	C-8	C-8a
**2a**	NMe_2	82.9145	51.8625	194.8822	115.3530	161.4857	112.0346	141.1071	110.7141	170.8434
**2b**	OMe	85.5306	53.6786	198.3672	115.5455	169.2519	105.4472	142.2540	113.9509	170.1887
**2c**	Me	85.1617	53.3574	202.6601	123.5281	151.8652	127.9101	141.3400	120.9697	170.0033
**1e**	H	86.2306	51.8539	200.8510	125.7788	132.6942	124.7722	142.4204	122.9802	168.7422
**2d**	Cl	86.2972	53.2214	199.4323	122.9977	149.3666	129.1123	140.7588	122.0479	170.4478
**2e**	$COCH_3$	85.4304	51.8561	201.1895	122.4214	153.3648	123.7140	142.7452	124.4841	168.2569
**2f**	NO_2	89.4624	52.2634	198.7606	119.2773	158.7295	122.1510	141.0412	127.7972	168.6527

[a] Values in parts per million (ppm).

Optimized structures

1a

C	1.92482900	-1.80370300	-0.09246500
C	0.55917400	-2.01231000	-0.18414600
C	-0.31916400	-0.91728000	-0.20359400
C	0.20253700	0.38458900	-0.13713500
C	1.59260600	0.58655100	-0.02829600
C	2.45600900	-0.50017800	-0.00673000
H	2.61471600	-2.64202400	-0.07619700
H	0.14744600	-3.01507700	-0.23668100
C	-0.70782600	1.54402100	-0.22504500
H	1.93879200	1.61203900	0.02318800
C	-2.15789800	1.19089300	-0.53154700
C	-2.59291400	-0.14663800	0.08881300
H	-2.80169300	2.00970800	-0.19690600
O	-1.65085700	-1.18928200	-0.30346300
O	-0.33177900	2.70264100	-0.11752700
H	-2.25124900	1.12642400	-1.62575700
C	-3.93287000	-0.61232500	-0.47640100
C	-2.63748000	-0.07849600	1.62203200
H	-4.72313800	0.10224800	-0.22340100
H	-3.87991500	-0.70473700	-1.56572400
H	-4.20121200	-1.58867000	-0.06022400
H	-2.92020300	-1.05304900	2.03207100
H	-1.66695100	0.20235200	2.04289400
H	-3.37470900	0.66323500	1.94808400
C	4.40075700	0.86557000	0.17387600
H	4.18200200	1.46555800	-0.71952500
H	4.05244100	1.40823000	1.06278700
H	5.47782400	0.70423300	0.24688300
O	3.81754800	-0.42518700	0.09003200

1b

C	-0.70954800	-1.13606600	0.71984400
C	0.56282500	-1.63949900	0.48791800
C	1.60360200	-0.77515400	0.11727900
C	1.34774700	0.60149300	-0.03020500
C	0.05618900	1.09946000	0.21115400
C	-0.96352900	0.23955300	0.58253900
H	-1.51411600	-1.80008500	1.02033400
H	0.77795500	-2.69675400	0.60453300
C	2.42674200	1.50468300	-0.48684000
H	-0.12648400	2.16295200	0.09961000
C	3.70996900	0.79672500	-0.90279900
C	3.99609100	-0.46058800	-0.06712500
H	3.59361200	0.50744900	-1.95767400
O	2.82528400	-1.33372600	-0.09478400
O	2.29315100	2.71524000	-0.57843200
H	4.54121100	1.50574200	-0.84810300
C	4.31438900	-0.11764600	1.39490700
C	5.11009000	-1.30022900	-0.68793100
H	5.22359200	0.49048400	1.45265000

H	3.50224000	0.44507900	1.86591100
H	4.47373100	-1.03576600	1.96880000
H	5.26558800	-2.21509900	-0.10716400
H	4.85354500	-1.58269000	-1.71380500
H	6.04827400	-0.73582200	-0.70475700
O	-2.19712500	0.77855200	0.90428600
C	-3.34303600	0.29831200	0.30031700
C	-4.53254600	0.47533900	1.01269700
C	-3.35616800	-0.28201900	-0.97124200
C	-5.73932800	0.06642800	0.44912000
H	-4.49027000	0.93333300	1.99592100
C	-4.57277400	-0.69303400	-1.51960100
H	-2.43159900	-0.40299500	-1.52553400
C	-5.76659600	-0.52349700	-0.81737300
H	-6.66203800	0.20596600	1.00605600
H	-4.58198700	-1.14275000	-2.50903700
H	-6.70842200	-0.84513200	-1.25225800

1c

C	-2.42576700	-1.47325600	-0.07283700
C	-1.08861300	-1.82880400	-0.18286100
C	-0.10255100	-0.83243600	-0.19565600
C	-0.48258500	0.51923500	-0.10584600
C	-1.84432700	0.84687800	0.01957400
C	-2.83423100	-0.12807000	0.03787000
H	-3.17875600	-2.25886300	-0.06373500
H	-0.78274100	-2.86786200	-0.25344400
C	0.54355900	1.57812400	-0.19192200
H	-2.09623500	1.90149700	0.09057000
C	1.94128300	1.07673100	-0.53427900
C	2.24496900	-0.30426300	0.06785600
H	2.00245600	1.01093800	-1.63066000
O	1.19023900	-1.24020500	-0.31113800
O	0.29763000	2.76781000	-0.05670100
H	2.67600100	1.81941500	-0.20976600
C	2.32515600	-0.25670200	1.60011700
C	3.51695200	-0.90299500	-0.52820100
H	3.14236400	0.39999800	1.91760900
H	1.39765900	0.12047500	2.04227200
H	2.51147400	-1.25965800	1.99656500
H	3.69138600	-1.90515600	-0.12355100
H	3.43250600	-0.98035200	-1.61672600
H	4.38190400	-0.27578400	-0.28819500
C	-4.29765800	0.22587400	0.16629400
H	-4.43691300	1.30790600	0.25083900
H	-4.87329500	-0.11717600	-0.70271500
H	-4.74788700	-0.23812000	1.05299700

1d

C	-1.43094900	-1.57515400	0.13523200
C	-0.08283200	-1.89230900	0.21772000
C	0.87460000	-0.86936800	0.21462500
C	0.44842200	0.46765600	0.13618700
C	-0.92545100	0.75566500	0.03887300

C	-1.89373800	-0.24321200	0.03649600
H	-2.14772800	-2.39211900	0.13982800
H	0.25191800	-2.92311300	0.27880100
C	1.44049300	1.55926700	0.20749700
H	-1.18907600	1.80536200	-0.02181300
C	2.85945900	1.10288700	0.52487800
C	3.19886200	-0.26563300	-0.08674700
H	3.56400200	1.86995700	0.19011700
O	2.18079200	-1.23738100	0.30329300
O	1.15435100	2.74103700	0.08030200
H	2.94176000	1.03690300	1.61986000
C	4.49841000	-0.82473200	0.48782800
C	3.25434800	-0.20981000	-1.61985600
H	5.33885200	-0.16908500	0.23702900
H	4.43304600	-0.90837000	1.57720600
H	4.69883200	-1.81938500	0.07677100
H	3.46845200	-1.20435600	-2.02341800
H	2.30800900	0.13745600	-2.04635100
H	4.04402800	0.47544900	-1.94659100
C	-3.40246800	0.04275000	-0.05903000
C	-4.10994300	-0.49275000	1.20842800
C	-3.98552000	-0.66242000	-1.30671700
C	-3.69937700	1.54959700	-0.17646400
H	-3.34301900	2.10509800	0.69819500
H	-3.23827300	1.98713600	-1.06902300
H	-4.78117100	1.70760900	-0.25086200
H	-5.18911900	-0.30399400	1.15329400
H	-3.96776200	-1.57205200	1.33059400
H	-3.72447900	-0.00104200	2.10882700
H	-3.51020500	-0.29431400	-2.22305300
H	-3.84188100	-1.74770200	-1.26874800
H	-5.06322400	-0.47328900	-1.38311900

1e

C	2.88404200	-1.30810400	-0.00467700
C	1.56679100	-1.72920200	-0.14467400
C	0.53471700	-0.78104400	-0.17248800
C	0.83788200	0.59170200	-0.06910700
C	2.17610700	0.99153700	0.08655400
C	3.19796300	0.05455300	0.11898900
H	3.67824000	-2.04993500	0.01706500
H	1.31089800	-2.78072600	-0.22677100
C	-0.24322000	1.59405700	-0.17374000
H	2.37545600	2.05583500	0.16767600
H	4.23073900	0.36936800	0.23415900
C	-1.60328600	1.01952300	-0.54982400
C	-1.84390700	-0.37715200	0.04321200
H	-2.38459000	1.72013200	-0.24100300
O	-0.73197200	-1.25335100	-0.31622700
O	-0.06409700	2.79383400	-0.02622600
H	-1.63532000	0.95321500	-1.64732100
C	-3.06847500	-1.04314400	-0.58020200
C	-1.95699200	-0.33976000	1.57356000
H	-3.97169500	-0.46615600	-0.35570500

H	-2.95839000	-1.11129800	-1.66705800
H	-3.19488900	-2.05507300	-0.18212500
H	-2.09420700	-1.35312700	1.96341100
H	-1.06131000	0.08758000	2.03520000
H	-2.81611000	0.26852500	1.87647200

1f

C	0.84518100	-1.71267900	0.12905000
C	-0.51787300	-1.97068000	0.10614000
C	-1.42491300	-0.91632100	-0.06463100
C	-0.93876400	0.39617300	-0.22394800
C	0.44354000	0.63130800	-0.18470800
C	1.36138000	-0.40499000	-0.00924100
H	1.53188800	-2.53918900	0.28964200
H	-0.90395900	-2.97652700	0.23719600
C	-1.88407600	1.50218100	-0.48821100
H	0.77564800	1.65450000	-0.33146900
C	-3.32393600	1.05990200	-0.71601300
C	-3.71518500	-0.15707700	0.13653300
H	-3.99143200	1.90574600	-0.52690000
O	-2.74660400	-1.22975600	-0.07898400
O	-1.54326400	2.67249800	-0.57170500
H	-3.41867700	0.79728300	-1.77999400
C	-5.04617000	-0.75091600	-0.31868000
C	-3.74426100	0.17693500	1.63445300
H	-5.85313100	-0.02255200	-0.18686200
H	-5.00033300	-1.03437800	-1.37491100
H	-5.28524100	-1.64396400	0.26771500
H	-3.98988300	-0.71865400	2.21345900
H	-2.77905800	0.55733300	1.98330600
H	-4.50185300	0.94141100	1.83813600
C	2.82340400	-0.14901400	0.02694400
C	3.33826000	1.00745900	0.63717300
C	3.73155200	-1.05437400	-0.54805400
C	4.71038700	1.25075500	0.66922300
H	2.65700100	1.70982300	1.10937600
C	5.10425900	-0.81352000	-0.51271600
H	3.35675100	-1.94171400	-1.05118400
C	5.60042200	0.34065900	0.09577400
H	5.08517900	2.14953900	1.15196200
H	5.78659600	-1.52482800	-0.97089700
H	6.67019800	0.52942100	0.12241800

1g

C	2.06673000	-1.54824800	-0.10227200
C	0.71910900	-1.86888800	-0.19607600
C	-0.24636800	-0.85163800	-0.20143600
C	0.15856800	0.49483400	-0.12122100
C	1.52300000	0.80871400	-0.01147200
C	2.46330400	-0.20676000	-0.00295400
H	2.81571600	-2.33329800	-0.09685500
H	0.39267900	-2.90172700	-0.25977200
C	-0.85065600	1.57483400	-0.20209200
H	1.80849000	1.85312000	0.05226300

C	-2.25745100	1.09662200	-0.53375100
C	-2.58229500	-0.27463100	0.07974700
H	-2.97698400	1.85393500	-0.20942100
O	-1.54495500	-1.23321400	-0.30071500
O	-0.57655900	2.75699100	-0.06937000
H	-2.32587600	1.02564200	-1.62931600
C	-3.86747300	-0.85504900	-0.50495500
C	-2.64947300	-0.21713000	1.61185400
H	-4.71935400	-0.21095700	-0.26344500
H	-3.79152600	-0.94091500	-1.59339500
H	-4.05703500	-1.85097500	-0.09215500
H	-2.85094700	-1.21401700	2.01579700
H	-1.71296100	0.14665500	2.04643800
H	-3.45273600	0.45577100	1.93058100
Cl	4.17533300	0.17709400	0.12751300

1h

C	-0.48794100	2.26910700	0.08707900
C	-1.86515400	2.14723300	0.12657200
C	-2.45047700	0.86983600	0.16006200
C	-1.62992100	-0.27714800	0.16929200
C	-0.23708600	-0.12705200	0.11644800
C	0.35365500	1.13432300	0.07445600
H	-0.01953600	3.24772500	0.05661700
H	-2.51408000	3.01716400	0.12261400
C	-2.23862300	-1.61881900	0.29159000
H	0.35241300	-1.03814600	0.12324300
C	-3.73631600	-1.60679700	0.56675000
C	-4.46147100	-0.45124700	-0.13986200
H	-4.16143200	-2.57237300	0.27805400
O	-3.80152700	0.81336300	0.19322300
O	-1.59377300	-2.65475300	0.23521000
H	-3.86587600	-1.50176500	1.65393700
C	-5.88619600	-0.28352600	0.38177600
C	-4.44559800	-0.61097800	-1.66588800
H	-6.47664200	-1.18035400	0.16740800
H	-5.88415200	-0.11687600	1.46348700
H	-6.36921400	0.57330400	-0.09858300
H	-4.93698200	0.24506500	-2.13815500
H	-3.42492200	-0.67963700	-2.05539200
H	-4.97931100	-1.52227600	-1.95634900
C	1.83456700	1.34979800	0.02311900
O	2.29182100	2.48400700	-0.00969600
C	4.23937900	0.48497000	-0.03937000
C	2.75160900	0.13110000	0.01346900
C	5.14138500	-0.75516000	-0.04019100
C	6.63739600	-0.41886000	-0.09328100
H	6.84131700	0.18109100	-0.99137300
H	6.89484700	0.22007200	0.76320500
H	4.93852900	-1.35777200	0.85809900
H	4.88493500	-1.39607800	-0.89735600
H	4.48661100	1.12815300	0.81418500
H	4.43380300	1.09080500	-0.93299000
H	2.53285600	-0.47551200	0.90479000

H	2.47724400	-0.50446700	-0.84148500
C	7.53438400	-1.66093700	-0.09295200
H	7.37670200	-2.26318500	0.81031800
H	7.32375600	-2.30205300	-0.95788300
H	8.59540600	-1.38888400	-0.13070700

1i

C	-1.27584200	-1.82597600	-0.11315700
C	0.09290400	-2.02208600	-0.17851500
C	0.95497300	-0.91320500	-0.18818000
C	0.42225100	0.39318700	-0.14667300
C	-0.96543100	0.56922200	-0.06792800
C	-1.82282400	-0.52818700	-0.04996000
H	-1.95587700	-2.67164700	-0.10105500
H	0.52324800	-3.01756000	-0.21318200
C	1.32644800	1.56035200	-0.24134400
H	-1.35015800	1.58222600	-0.03523300
C	2.77570600	1.20996900	-0.55178200
C	3.22283300	-0.10482800	0.10522800
H	2.86083300	1.11388700	-1.64426700
O	2.28211700	-1.16965000	-0.24967900
O	0.94403500	2.71514800	-0.13646600
H	3.41776800	2.04079600	-0.24527100
C	3.26763400	0.00425100	1.63507500
C	4.56142700	-0.58230200	-0.45188700
H	4.00381800	0.75627800	1.93855800
H	2.29730700	0.29577900	2.04947000
H	3.55306100	-0.95762300	2.07192400
H	4.83612300	-1.54443300	-0.00801000
H	4.50548900	-0.70616100	-1.53785000
H	5.34862600	0.14333200	-0.22249100
C	-3.30150700	-0.39760100	0.02609600
O	-4.07463100	-1.33687300	0.03978300
O	-3.70481700	0.89254100	0.07945900
C	-5.12461200	1.08848300	0.15186500
H	-5.53320700	0.61418700	1.04822500
H	-5.26795900	2.16874800	0.18950100
H	-5.61645900	0.66518800	-0.72812000

1j

C	2.17763900	-1.56364000	-0.09523800
C	0.83309100	-1.87874700	-0.18501300
C	-0.12847000	-0.85409400	-0.18820600
C	0.28029800	0.49414000	-0.11459500
C	1.64182600	0.79882400	-0.01076100
C	2.59877800	-0.21754100	0.00059600
H	2.92089700	-2.35462800	-0.08968700
H	0.49805300	-2.90878900	-0.24517100
C	-0.72768600	1.57727000	-0.20241200
H	1.93039700	1.84326000	0.04727200
C	-2.13081600	1.10026600	-0.54701000
C	-2.46626100	-0.26225500	0.07924900
H	-2.85307400	1.86189300	-0.23973000
O	-1.42278300	-1.23047800	-0.27536700

O	-0.44990800	2.75717200	-0.06597500
H	-2.18598000	1.01703900	-1.64242200
C	-3.74383500	-0.84990900	-0.51369000
C	-2.54743900	-0.18832900	1.60935300
H	-4.59745200	-0.20188900	-0.28989900
H	-3.65580700	-0.94894400	-1.60004600
H	-3.93949700	-1.84018900	-0.09056200
H	-2.75200400	-1.18052900	2.02284300
H	-1.61599800	0.18234100	2.04925900
H	-3.35460100	0.48720700	1.91181500
C	3.99364100	0.09239700	0.10196600
N	5.12885500	0.33419800	0.18452000

1k

C	1.82740600	-1.57798200	-0.10331900
C	0.48230400	-1.89238900	-0.18343500
C	-0.47974200	-0.86596500	-0.18639300
C	-0.07282000	0.48536900	-0.12352800
C	1.28842000	0.79283400	-0.02914400
C	2.21951100	-0.23375700	-0.01919100
H	2.58648700	-2.35064600	-0.09556500
H	0.14605700	-2.92233300	-0.23555300
C	-1.08357500	1.56630000	-0.21486200
H	1.59837100	1.82990100	0.02142700
C	-2.48576300	1.08352400	-0.55552900
C	-2.81879400	-0.27345500	0.08301500
H	-3.20954100	1.84619800	-0.25466800
O	-1.77188700	-1.24372100	-0.26186900
O	-0.80814900	2.74679300	-0.08433300
H	-2.54096700	0.99062200	-1.65013100
C	-4.09411100	-0.87041500	-0.50512100
C	-2.89917500	-0.18676500	1.61223400
H	-4.94942700	-0.22252500	-0.28770600
H	-4.00536100	-0.97919300	-1.59046500
H	-4.28744300	-1.85713800	-0.07277600
H	-3.10069900	-1.17577100	2.03468100
H	-1.96885200	0.19099600	2.04850500
H	-3.70835500	0.48900200	1.90857400
N	3.64595300	0.08696600	0.07429100
O	3.96381100	1.27358700	0.15035600
O	4.44323900	-0.85311600	0.07150500

2a

C	-2.16565500	0.03987000	-0.04609300
C	-1.06558100	-0.83633000	-0.16093900
C	0.23717000	-0.34284100	-0.18363500
C	0.49450900	1.04124200	-0.08922900
C	-0.60764600	1.90359800	0.05063500
C	-1.90505100	1.43548800	0.07373800
H	-1.19337000	-1.90905100	-0.22303700
C	1.86248800	1.55556400	-0.18742400
H	-0.40263600	2.96729300	0.12857700
C	2.91129000	0.49910700	-0.52386300
C	2.58471400	-0.88042600	0.06848200

H	2.95379300	0.41809900	-1.61987000
O	1.22964700	-1.26651700	-0.31336200
O	2.15910100	2.73882700	-0.06682300
H	3.89064500	0.85067200	-0.18521000
C	2.67852600	-0.88221600	1.60097100
C	3.47091200	-1.96908000	-0.53353200
H	3.70202800	-0.65627600	1.91953600
H	2.01342200	-0.13539300	2.04547400
H	2.40224700	-1.86640400	1.99253100
H	3.19300800	-2.95066000	-0.13584400
H	3.36181700	-1.99499600	-1.62240200
H	4.52245800	-1.78133400	-0.29212100
H	-2.72113100	2.13951100	0.17684500
N	-3.45686400	-0.44190800	-0.04127600
C	-3.69896700	-1.87312300	-0.11103300
C	-4.57469600	0.46131400	0.18124300
H	-3.28573300	-2.40735300	0.75729600
H	-4.77453300	-2.05448900	-0.14206900
H	-3.25783500	-2.30777000	-1.01717700
H	-4.62887300	1.23462400	-0.59599900
H	-5.50499800	-0.10805000	0.15049200
H	-4.51432300	0.96344800	1.15745900

2b

C	2.48073700	0.17028000	0.01346100
C	1.45043900	-0.76137400	-0.12432100
C	0.12271400	-0.31504900	-0.16081000
C	-0.17964500	1.05685600	-0.06649600
C	0.88206200	1.97044500	0.08895500
C	2.19403600	1.54834000	0.12883700
H	1.63396600	-1.82596800	-0.19528500
C	-1.56975900	1.51925400	-0.18535200
H	0.62936100	3.02371400	0.16288700
C	-2.56960000	0.42764700	-0.55213400
C	-2.20832700	-0.94043500	0.04563000
H	-3.56989100	0.74148600	-0.23911500
O	-0.82863600	-1.27492200	-0.30382900
O	-1.90901200	2.68844800	-0.05934400
H	-2.57884600	0.34984000	-1.64918800
C	-3.03799900	-2.05928300	-0.58001700
C	-2.33601200	-0.94935500	1.57526600
H	-4.10123400	-1.91029900	-0.36466000
H	-2.90113800	-2.07880400	-1.66582200
H	-2.73474700	-3.03075700	-0.17635900
H	-2.03409100	-1.92389000	1.97163400
H	-1.70876800	-0.18037300	2.03683000
H	-3.37388800	-0.76080300	1.87043200
H	3.01955700	2.24317100	0.23972800
C	4.16812800	-1.52096100	-0.06248000
H	3.83829000	-1.94144300	-1.02081500
H	5.25802900	-1.53977800	-0.01315600
H	3.75557800	-2.12145700	0.75815400
O	3.79854400	-0.15387800	0.05836000

2c

C	-2.76700100	-0.47513500	-0.01362500
C	-1.57440000	-1.18222200	-0.15165500
C	-0.34333000	-0.51248300	-0.17553500
C	-0.30105400	0.89053500	-0.06833900
C	-1.50879300	1.59454300	0.08555400
C	-2.72181500	0.92972400	0.11254700
H	-1.57320700	-2.26498000	-0.23577800
C	0.98723300	1.60115800	-0.16960500
H	-1.45257700	2.67576900	0.16919600
C	2.16908100	0.71414700	-0.54294600
C	2.06123600	-0.70209900	0.04317600
H	2.19145200	0.64749000	-1.64064000
O	0.76946900	-1.28067100	-0.31823000
O	1.10510700	2.80934200	-0.02311500
H	3.09544500	1.20235100	-0.22623800
C	2.18020000	-0.69979600	1.57370400
C	3.08722800	-1.64357300	-0.58357500
H	3.16077700	-0.31755100	1.87733200
H	1.41401500	-0.07082700	2.03777400
H	2.06909700	-1.71794000	1.95948700
H	2.96592000	-2.65689500	-0.18732500
H	2.96222500	-1.68118900	-1.67029600
H	4.10349900	-1.30246000	-0.35995800
H	-3.64784400	1.48762800	0.22616200
C	-4.09302000	-1.19673200	0.01133100
H	-4.74250900	-0.85583100	-0.80451100
H	-3.96617600	-2.27865000	-0.08912500
H	-4.63011000	-1.00468600	0.94849300

2d

C	2.44505100	-0.12480000	-0.01765400
C	1.35292200	-0.97115100	-0.15472100
C	0.06639400	-0.41499300	-0.17710200
C	-0.10546700	0.97985500	-0.06981200
C	1.02498000	1.79848200	0.08301800
C	2.30364200	1.26442100	0.11015300
H	1.47306400	-2.04490400	-0.23628200
C	-1.45819800	1.56575900	-0.16949600
H	0.86414300	2.86898000	0.16633900
C	-2.54987600	0.57181600	-0.54387600
C	-2.31012700	-0.82748700	0.04422000
H	-3.51845400	0.97044700	-0.22849800
O	-0.96660500	-1.28161000	-0.31797900
O	-1.68489800	2.75673100	-0.01931200
H	-2.56443300	0.50294700	-1.64152900
C	-3.24001100	-1.86317700	-0.58270000
C	-2.42626900	-0.83530700	1.57441800
H	-4.28396500	-1.62219400	-0.35681400
H	-3.11397400	-1.88711200	-1.66959400
H	-3.02056200	-2.86019500	-0.18772400
H	-2.21155600	-1.83604600	1.96163000
H	-1.72928200	-0.13025100	2.03854200
H	-3.44069000	-0.55535200	1.87795300

H	3.17863600	1.89451500	0.22053900
Cl	4.05652000	-0.82131300	0.01029800

2e

C	2.19530800	-0.02923400	-0.02969600
C	1.10311400	-0.89007400	-0.15228300
C	-0.19773100	-0.37892500	-0.17523600
C	-0.40735700	1.01301200	-0.08451000
C	0.69791600	1.86698700	0.05354600
C	1.98788000	1.36078200	0.08077500
H	1.26827600	-1.95919100	-0.22406500
C	-1.77969400	1.56066900	-0.18390600
H	0.50518800	2.93303600	0.12495700
C	-2.84315400	0.53388500	-0.54612800
C	-2.56291200	-0.85083200	0.05963300
H	-3.82283400	0.90994500	-0.23726900
O	-1.21151400	-1.27326200	-0.30225700
O	-2.03666300	2.74591900	-0.04051700
H	-2.85358700	0.45023800	-1.64292700
C	-3.46913600	-1.91804500	-0.54914500
C	-2.67423900	-0.84013900	1.59049800
H	-4.51799900	-1.70270600	-0.31995000
H	-3.34819500	-1.95170700	-1.63639600
H	-3.22005800	-2.90387400	-0.14399200
H	-2.43239100	-1.82964200	1.99021000
H	-1.99344700	-0.11181000	2.04281800
H	-3.69423900	-0.58157900	1.89456100
H	2.82875000	2.03827900	0.18197000
C	3.56894300	-0.64537400	-0.01078600
O	3.70651800	-1.85458600	-0.10258200
C	4.77730700	0.26509500	0.12397600
H	4.73498100	0.84108800	1.05614300
H	5.67959700	-0.34846100	0.11839000
H	4.82175400	0.98498200	-0.70202800

2f

C	2.20787400	0.00492900	-0.02164300
C	1.15346300	-0.88735400	-0.14476800
C	-0.15073300	-0.37638500	-0.17019900
C	-0.36248300	1.01517100	-0.08127200
C	0.73582700	1.87843000	0.05793300
C	2.03205100	1.38848000	0.08853100
H	1.33023400	-1.95269500	-0.21372400
C	-1.74064100	1.55592500	-0.18404500
H	0.53573300	2.94284600	0.12767400
C	-2.79577800	0.52497600	-0.55500600
C	-2.51558400	-0.85781000	0.05497200
H	-3.77952700	0.89653600	-0.25429500
O	-1.15572700	-1.27448600	-0.29640900
O	-1.99967300	2.73873600	-0.03586100
H	-2.79663100	0.44095600	-1.65177900
C	-3.40965500	-1.93055300	-0.56084000
C	-2.63559200	-0.84655400	1.58452800
H	-4.46128400	-1.71988400	-0.34082200

H	-3.27958500	-1.96494700	-1.64692300
H	-3.15969000	-2.91443500	-0.15176800
H	-2.39153100	-1.83419600	1.98718500
H	-1.96287600	-0.11353400	2.04158700
H	-3.65902800	-0.59344900	1.88097600
H	2.89371200	2.03541800	0.18755700
N	3.58186100	-0.53977200	0.00512800
O	4.50722900	0.26150300	0.12248800
O	3.71629000	-1.75822000	-0.09155800

2g

C	2.76271600	-0.52537600	-0.01133900
C	1.56614600	-1.22890200	-0.14939200
C	0.35463500	-0.53053100	-0.17480700
C	0.34018200	0.87553500	-0.06676200
C	1.56411400	1.55347700	0.08826300
C	2.76811700	0.87677600	0.11662600
H	1.55035200	-2.31285100	-0.22792300
C	-0.92841500	1.61194100	-0.17033600
H	1.52831000	2.63538000	0.17177000
C	-2.13128800	0.74996300	-0.53880600
C	-2.05321900	-0.66917700	0.04395100
H	-3.04473900	1.25751200	-0.21503400
O	-0.77213000	-1.27436700	-0.31976000
O	-1.02220400	2.82392600	-0.03039200
H	-2.16268600	0.68664400	-1.63636800
C	-3.09716300	-1.58899000	-0.58507200
C	-2.17057500	-0.66895100	1.57427600
H	-4.10620700	-1.22663600	-0.36238500
H	-2.97192500	-1.62791600	-1.67171300
H	-2.99820900	-2.60509900	-0.18958200
H	-2.07846900	-1.68987400	1.95786600
H	-1.39270000	-0.05477900	2.03854800
H	-3.14373600	-0.26941200	1.87934500
H	3.71612200	1.39174800	0.22784500
O	3.97101300	-1.15111200	0.01911900
H	3.83895800	-2.10688700	-0.08371100

3a

C	-1.17244200	2.74519100	0.01082400
C	0.19731300	2.51162900	-0.00948800
C	0.65461200	1.19386900	0.04172400
C	-0.24128700	0.09119500	0.11177300
C	-1.65437600	0.35746000	0.02123800
C	-2.08852800	1.69740100	0.02008200
H	0.92170300	3.31716000	-0.05534300
C	0.31138700	-1.20853400	0.53764400
C	1.81570800	-1.22245100	0.80744200
C	2.59688500	-0.25789900	-0.08355200
H	1.96690700	-0.94184600	1.85962800
O	2.00816500	1.06277500	0.06579000
O	-0.34447200	-2.21838900	0.76919700
H	2.16964600	-2.25108500	0.69239500
C	2.55291200	-0.65771700	-1.56565400

C	4.03953900	-0.09163500	0.39052500
H	3.02993200	-1.63321000	-1.71086100
H	1.52531500	-0.72380200	-1.93533500
H	3.08731000	0.08280600	-2.16908200
H	4.55761300	0.65533600	-0.21984800
H	4.06616900	0.23730800	1.43410900
H	4.57854100	-1.04143900	0.30880900
H	-3.14646600	1.92119900	-0.04079500
H	-1.53982900	3.76795700	-0.02256600
N	-2.58194700	-0.67096200	-0.03105900
C	-3.96733300	-0.40583400	0.31289300
C	-2.42428000	-1.78349400	-0.96306300
H	-4.45334600	-1.35868400	0.54807400
H	-4.02520300	0.23068900	1.19940000
H	-4.53839100	0.07032600	-0.50340800
H	-3.19056300	-1.71177000	-1.75147600
H	-1.44588700	-1.74855700	-1.43827300
H	-2.52001800	-2.74582600	-0.45225000

3b

C	1.63969500	2.44547100	-0.02410200
C	0.25914800	2.35836000	-0.11275500
C	-0.34177500	1.09390800	-0.15199000
C	0.42956300	-0.09224000	-0.11146200
C	1.84609500	0.03732800	-0.00125700
C	2.44127100	1.30201200	0.03762500
H	-0.37363400	3.23835800	-0.14398200
C	-0.25990300	-1.39745700	-0.23820700
C	-1.73686000	-1.29265600	-0.61568800
C	-2.43890600	-0.11414400	0.06255400
H	-1.79533300	-1.16378800	-1.70618400
O	-1.69750600	1.10194800	-0.24390200
O	0.26568000	-2.49118900	-0.10510300
H	-2.22076800	-2.24135500	-0.36714200
C	-2.50216600	-0.27625000	1.58789900
C	-3.82948000	0.12826000	-0.52005300
H	-3.09332500	-1.15993700	1.85162800
H	-1.50492900	-0.39567500	2.02261400
H	-2.97141300	0.60349200	2.03934700
H	-4.28443700	1.01406400	-0.06475100
H	-3.77179600	0.28764700	-1.60142600
H	-4.47768800	-0.73301900	-0.32732400
H	3.51590400	1.40673200	0.11509400
H	2.11268400	3.42352400	0.00904000
C	3.97111500	-1.04483200	0.11886600
H	4.31104700	-2.08144900	0.13444600
H	4.39732700	-0.53359400	-0.75395500
H	4.30348900	-0.53843200	1.03419400
O	2.55508700	-1.11240100	0.04523300

3c

C	0.00000000	0.00000000	0.00000000
C	0.00000000	0.00000000	1.38656880
C	1.22146446	0.00000000	2.06981387

C	2.45100194	0.01057460	1.36623809
C	2.43420702	-0.00869782	-0.05770154
C	1.20337793	-0.01127484	-0.71341765
H	-0.91869657	-0.00830400	1.96370375
C	3.70277010	0.09207501	2.15065358
C	3.51833123	0.34330427	3.64353425
C	2.29202813	-0.37332152	4.21615736
H	4.43452392	0.04505104	4.16106798
O	1.12785041	0.00294468	3.42744661
O	4.82664512	0.01791656	1.67056955
H	3.39650718	1.42747088	3.78324552
C	1.96946874	0.10474367	5.63029390
C	2.44232527	-1.90050890	4.17284708
H	2.79114637	-0.14007685	6.31148948
H	1.81435859	1.18814598	5.64453713
H	1.05834085	-0.37876084	5.99734835
H	1.53111269	-2.37737841	4.54708873
H	2.62489247	-2.26006195	3.15534958
H	3.28327963	-2.21702830	4.79928746
H	1.18377534	-0.01972092	-1.79960342
H	-0.94515641	-0.00142390	-0.53684457
C	3.69872669	-0.01266378	-0.88295039
H	4.31338378	-0.89357440	-0.67510822
H	3.44912525	0.00093655	-1.94882088
H	4.33294947	0.84993584	-0.65734009

3d

C	-1.68081700	2.51429800	-0.00177500
C	-0.30252400	2.39684100	-0.09383900
C	0.28181000	1.12519600	-0.14118000
C	-0.50971700	-0.05288100	-0.10301300
C	-1.91050500	0.11755700	0.00733100
C	-2.49361000	1.37867600	0.05432900
H	0.34713300	3.26484500	-0.12434600
C	0.18043300	-1.36665900	-0.22548700
C	1.64562800	-1.27165600	-0.63526100
C	2.36951800	-0.10909400	0.04812100
H	2.12547800	-2.22810700	-0.41102500
O	1.63564400	1.11777900	-0.23833500
O	-0.34961100	-2.45026400	-0.05397600
H	1.68181800	-1.12461400	-1.72440300
C	3.75483700	0.12414700	-0.54951400
C	2.44728900	-0.28670900	1.57061300
H	4.39625500	-0.74506600	-0.37088500
H	3.68641700	0.29286500	-1.62875600
H	4.22376800	1.00130000	-0.09202900
H	2.93390700	0.58194400	2.02475400
H	1.45426500	-0.39701500	2.01770500
H	3.02917600	-1.18080900	1.81881900
H	-3.57114000	1.46652900	0.13202100
H	-2.13672600	3.49971900	0.03623300
Cl	-3.00898100	-1.24647100	0.06343800

3e

C	-1.15529700	2.78544600	-0.06956400
C	0.20531600	2.51994200	-0.00454100
C	0.64344900	1.19344900	0.10444400
C	-0.28835700	0.13286000	0.14379000
C	-1.67005800	0.42210900	0.04022900
C	-2.09377200	1.74484600	-0.04688700
H	0.94571800	3.31268000	-0.03023200
C	0.19273500	-1.23441400	0.41605600
C	1.68245900	-1.35651500	0.70116300
C	2.52558400	-0.32572800	-0.06055100
H	1.81425100	-1.20323900	1.78224900
O	1.98536500	1.00703300	0.19348500
O	-0.55225400	-2.20347400	0.48397200
H	2.00322400	-2.37810300	0.47702100
C	2.51499000	-0.57619600	-1.57478100
C	3.95395700	-0.26546800	0.47510700
H	2.96203300	-1.55028100	-1.80081500
H	1.49820900	-0.56913300	-1.98013100
H	3.09369200	0.19856200	-2.08696300
H	4.52329400	0.51074100	-0.04607200
H	3.95467200	-0.03419600	1.54484100
H	4.45794500	-1.22590300	0.32465400
H	-3.15580500	1.96367600	-0.09580300
H	-1.49595800	3.81475400	-0.14391400
O	-3.56706600	-0.67816500	0.93394300
C	-2.74846400	-0.64837400	0.03494700
C	-2.88226000	-1.51034800	-1.20624500
H	-3.47024900	-0.95047900	-1.94717900
H	-1.91736700	-1.75662300	-1.65225100
H	-3.42369600	-2.42526300	-0.95560400

3f

C	-1.33122500	2.69370400	0.00033600
C	0.03348500	2.48078300	-0.11676600
C	0.53863300	1.17041100	-0.15666300
C	-0.33258800	0.06177500	-0.09547300
C	-1.70945700	0.33064900	0.00325500
C	-2.22188500	1.61432500	0.07203900
H	0.73658500	3.30553700	-0.16412700
C	0.24987900	-1.30623800	-0.06934100
C	1.68344600	-1.36343600	-0.56834400
C	2.52746800	-0.22673300	0.03071400
H	2.10676600	-2.33921300	-0.31622500
O	1.88404800	1.05557900	-0.25765300
O	-0.34990100	-2.28369400	0.33819300
H	1.67175300	-1.26612200	-1.66316500
C	3.89320400	-0.12943800	-0.64369100
C	2.66603300	-0.35515800	1.55308800
H	4.46838100	-1.04377800	-0.46554400
H	3.78228000	0.00962400	-1.72350300
H	4.45616500	0.71861100	-0.24119000
H	3.24042400	0.48778100	1.94908800
H	1.69054300	-0.37047800	2.04994200
H	3.18732400	-1.28346700	1.80971600

H	-3.29152100	1.76538300	0.15804800
H	-1.71615600	3.70838500	0.03931500
N	-2.70366500	-0.76282800	-0.06316400
O	-2.68307900	-1.47518000	-1.05952000
O	-3.52467400	-0.81874500	0.84833700

References

[S1] P. Morales, L. M. Azofra, J. Cumella, L. Hernandez-Folgado, M. Roldán, I. Alkorta, N. Jagerovic, *ARKIVOC* **2014**, *2014*, 319–332.
